# Biomimetic Scaffolds Enhance iPSC Astrocyte Progenitor Angiogenic, Immunomodulatory, and Neurotrophic Capacity in a Stiffness and Matrix‐Dependent Manner for Spinal Cord Repair Applications

**DOI:** 10.1002/adhm.202500830

**Published:** 2025-05-19

**Authors:** Cian O'Connor, Ian Woods, Sarah F. McComish, Sean Kerr, Matthew McGrath, Juan Carlos Palomeque Chávez, Jack Maughan, Tara McGuire, Maeve A. Caldwell, Adrian Dervan, Fergal J. O'Brien

**Affiliations:** ^1^ Tissue Engineering Research Group Department of Anatomy & Regenerative Medicine Royal College of Surgeons in Ireland (RCSI) Dublin 2 Ireland; ^2^ Advanced Materials and Bioengineering Research Centre (AMBER) RCSI and Trinity College Dublin (TCD) Dublin 2 Ireland; ^3^ Trinity Centre for Biomedical Engineering Trinity College Dublin (TCD) Dublin 2 Ireland; ^4^ Department of Physiology TCD Dublin 2 Ireland; ^5^ Trinity College Institute of Neuroscience TCD Dublin 2 Ireland; ^6^ School of Physics TCD Dublin 2 Ireland

**Keywords:** astrocytes, induced pluripotent stem cells, neurons, scaffold, spinal cord injury

## Abstract

Spinal cord injury repair poses a significant challenge due to the hostile microenvironment of the injury site and the poor survival and function of clinically relevant transplanted cells. Here it is aimed to investigate whether tuning the physicochemical properties of implantable biomimetic biomaterial scaffolds can enhance the localized delivery and reparative potential of patient‐derived induced pluripotent stem cells (iPSC) astrocyte progenitors. It is demonstrated that soft, collagen‐IV/fibronectin‐functionalized hyaluronic acid scaffolds, mimicking the physicochemical properties of healthy spinal cord tissue, optimally support the formation of iPSC‐derived multicellular spheroids, promoting neural cell survival and function. These soft, collagen‐IV/fibronectin scaffolds enhance angiogenic cytokine release, facilitate vascular network formation, modulate inflammatory responses, and promote neurite outgrowth from growing, mature and injured neurons, while supporting cell infiltration from spinal cord explants. These findings demonstrate that optimized biomimetic scaffold properties provide a supportive environment for iPSC astrocyte progenitors but can also modulate their reparative capacity. These findings highlight the critical role of matrix composition and scaffold stiffness in advancing scaffold‐mediated patient‐derived stem cell‐delivery strategies for spinal cord repair applications.

## Graphical abstract

Through tuning biomimetic scaffold stiffness and matrix composition the reparative capacity of astrocyte progenitors is enhanced. Soft, collagen‐IV/fibronectin‐functionalized scaffolds promote progenitor growth while improving angiogenic, immunomodulatory, and neurotrophic capacity in a stiffness and matrix‐dependent manner, demonstrating the impact of tuning scaffold properties on stem‐cell‐based approaches for spinal cord repair applications

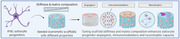

## 1 Introduction

Spinal cord injury (SCI) is a complex pathophysiology defined by the development of an unsupportive environment for the regrowth of injured axons through the lesion site, attenuating recovery for the patient.^[^ ^1^ ^]^ Failure of the injured cord to regenerate is in part due to the poor intrinsic capacity of neurons to regrow their damaged axons,^[^ ^2^ ^]^ the inhibitory environment created by injury‐responsive glia^[^ ^3^, ^4^ ^]^ and the extensive loss of tissue suffered with the formation of a lesion cavity.^[^ ^2^, ^5^, ^6^ ^]^ Astrocytes under healthy conditions provide trophic support to neurons^[^ ^7^ ^]^ but following injury adopt a reactive phenotype and over time, become scar‐forming.^[3,^ ^7^, ^8^ ^]^ Axonal damage from the initial injury and concurrent secondary injury also results in extensive cell death and contributes to lesion cavity formation. In addition, the initial trauma, subsequent infiltration of immune cells, and excessive inflammation can damage the local vasculature leading to ischemic damage.^[^ ^9^ ^]^ The various challenges associated with SCI present a need for a therapeutic approach to promote axonal regrowth through the lesion site, attenuate inflammation, and form stable vascular networks necessary for the restoration of sensorimotor control.^[^ ^9^, ^10^, ^11^, ^12^, ^13^ ^]^ Different approaches have been employed to tackle individual facets of SCI but no one strategy is available to fully promote repair, largely due to the complex nature of SCI pathophysiology.^[^ ^13^, ^14^ ^]^ Therefore, the need for developing a multifaceted approach capable of bridging the lesion, promoting axonal growth, replacing lost cells, encouraging angiogenesis, and positively influencing glial cell behavior is ideally required.

The delivery of stem or progenitor cells directly to the lesion site has been widely explored as an avenue for SCI repair to encourage axonal growth and offer support via trophic paracrine signaling.^[^ ^11^ ^]^ The delivery of neural progenitor cells^[^ ^15^, ^16^, ^17^ ^]^ and/or astrocyte precursors^[^ ^18^, ^19^ ^]^ can promote axonal regrowth and reduce secondary inflammatory‐mediated damage through paracrine activity.^[^ ^15^, ^17^ ^]^ Induced pluripotent stem cell (iPSC)‐derived progenitors offer the advantage of a noninvasive, clinically relevant, and patient‐specific cell resource that can be reprogrammed to desired cell populations from donor cells.^[^ ^20^, ^21^ ^]^ In particular, iPSC‐derived astrocyte progenitors might offer support through the release of supportive cytokines at the injury site,^[^ ^22^ ^]^ encouraging axonal growth,^[^ ^18^ ^]^ vascular network formation, and dampening of host‐tissue inflammation. However, while transplanted iPSC astrocyte progenitors may offer enhanced support to promote axonal regrowth, due to the harsh environment of the injured cord, where inflammation can persist from months to years,^[^ ^3^, ^23^ ^]^ transplanted cells typically exhibit poor survival and integration.^[^ ^24^ ^]^

Biomaterial‐based scaffolds designed for tissue engineering applications offer a promising approach to promote recovery following SCI by physically bridging the lesion site with a supportive 3D framework capable of supporting cord resident glia and re‐growing axons across the injury site.^[^ ^14^, ^24^, ^25^ ^]^ Similarly, scaffolds can deliver cells, which can replace lost tissue and offer support via paracrine signaling (i.e., release of supportive cytokines) to encourage axonal regrowth.^[^ ^14^, ^18^, ^24^ ^]^ However, clinical translation of tissue‐engineered scaffolds for SCI repair has been limited in part by the use of unoptimized mixes of proteins or synthetic biomaterials that lack proteins and do not mimic the native spinal cord physicochemical properties,^[^ ^14^, ^26^ ^]^ thus, potentially inducing inflammation and further astrocyte‐mediated scarring^[^ ^27^, ^28^ ^]^ in the spinal cord environment.^[^ ^14^, ^27^, ^28^, ^29^ ^]^ Previous research has demonstrated that by studying interactions with individual extracellular matrix constituents, it is possible to enhance axonal outgrowth^[^ ^30^, ^31^, ^32^, ^33^, ^34^ ^]^ and alter resting and injured astrocyte responses.^[^ ^29^, ^33^, ^35^ ^]^ However, less is known about the effectiveness of extracellular matrix molecule combinations to enhance neuronal outgrowth,^[^ ^36^ ^]^ induce resting astrocyte phenotypes,^[^ ^35^ ^]^ and how they can be effectively incorporated into scaffolds to support cell delivery of clinically relevant patient‐derived stem cells.^[^ ^37^, ^38^ ^]^ Similarly, we and others have previously demonstrated that scaffold stiffness can dictate neural cell responses,^[^ ^27^, ^39^, ^40^ ^]^ where scaffolds matching the stiffness of the healthy and injured spinal cord (<1.5 kPa) can promote neuronal growth and morphologies typical of resting astrocytes, compared to stiffer scaffolds.^[^ ^33^ ^]^ However, while it has been reported that scaffold physicochemical properties can affect the biocompatability^[^ ^41^ ^]^ and/or differentiation^[^ ^42^ ^]^ of cells, less is known about whether properties such as scaffold stiffness or matrix composition can enhance the reparative potential of patient derived cell cargoes.^[^ ^43^ ^]^

Building off our previous work on investigating whether scaffold properties influence the growth of cells of the surrounding spinal cord environment, this study aimed to investigate whether tuning the physicochemical properties of previously developed biomimetic scaffold^[^ ^33^ ^]^ could support the delivery of iPSC astrocyte progenitor cells and enhance their reparative potential for spinal cord repair applications.^[^ ^24^ ^]^ The results demonstrate that soft, biomimetic collagen‐IV (CIV and fibronectin (FN)‐functionalized hyaluronic acid (HyA) scaffolds mimicking the stiffness and matrix composition of the healthy spinal cord could effectively support iPSC astrocyte progenitor delivery. Specifically, soft (<1.5 kPa) CIV/FN‐functionalized HyA scaffolds promoted iPSC astrocyte progenitor infiltration, self‐assembly into large multicellular spheroid structures, scaffold colonization, and displayed enhanced glutamate uptake function. Furthermore, these soft biomimetic scaffolds significantly enhanced the capacity of iPSC astrocyte progenitors to promote neurite outgrowth from spinal cord neurons, and encouraged vascular network formation and ameliorated inflammatory responses of injured astrocytes in a stiffness and matrix‐dependent manner. Finally, astrocyte infiltration from spinal cord explants and axonal extensions between dorsal root ganglia (DRGs) and progenitor spheroids were observed within scaffolds mimicking the stiffness of the healthy spinal cord, demonstrating the importance of optimizing scaffold physicochemical properties for enhancing the effectiveness of patient‐derived cell delivery strategies for SCI and neural applications more broadly.

## 2 Results

### 2.1 Collagen‐IV and Fibronectin Promote Robust Outgrowth from iPSC Neurons and Astrocytes

**Figure 1:**
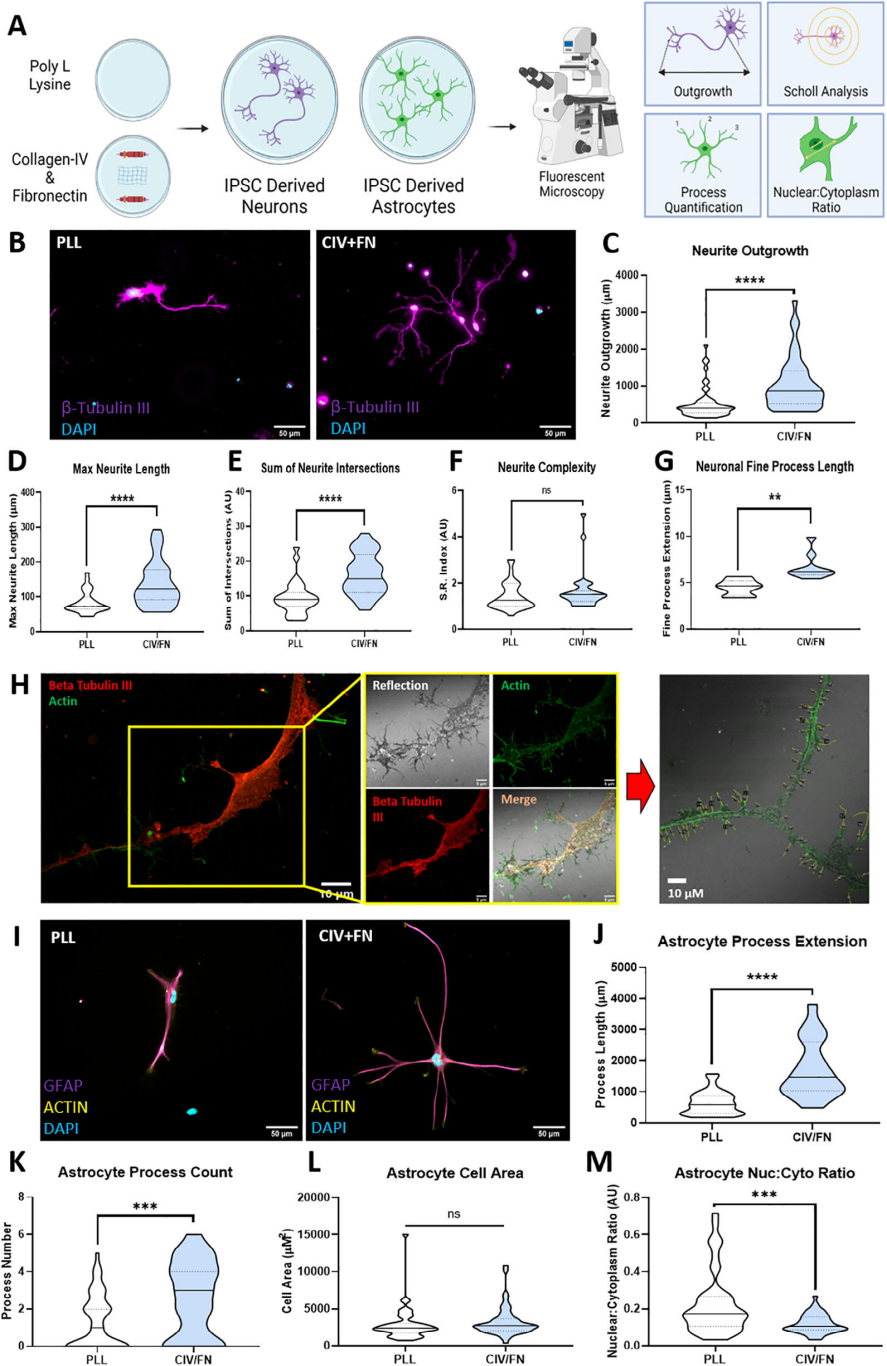
Collagen‐IV and fibronectin CIV/FN substrates significantly enhanced iPSC‐derived neuronal and astrocyte outgrowth. A) Experimental outline of analyzing iPSC‐derived neuron and astrocyte morphological features when cultured on poly‐l‐lysine controls (PLL) and CIV/FN substrates. B) Immunofluorescent staining of iPSC‐derived neurons cultured on both PLL and CIV/FN substrates revealed distinct morphological differences. C) CIV/FN substrates significantly enhanced neurite outgrowth of iPSC‐derived neurons compared to neurons grown on PLL only. Scholl analysis showed that CIV/FN substrates promoted D) max neurite length and E) the number of intersections/branching points. F) Neurite complexity was not affected by CIV/FN substrates. G,H) Confocal microscopy paired with reflection imaging revealed that CIV/FN substrates significantly increased fine neurite process extension. I) Immunostaining of iPSC‐derived astrocytes on PLL and CIV/FN substrates also showed distinct morphologies where J) process extension and K) process count were enhanced compared to astrocytes grown on PLL substrates. L) CIV/FN substrates did not affect the overall astrocyte cell area. M) Nuclear:cytoplasmic ratio was decreased in astrocytes cultured on CIV/FN substrates when compared with PLL substrates. All analyses via unpaired two‐tailed *t*‐test. For neuronal data, *N* = 3, *n* = 31–52 fields of view. For astrocyte data, *N* = 3, *n* = 41–44 fields of view.

Previous work from our lab has identified extracellular matrix molecules CIV and FN as potent neurotrophic substrates to support both neurons and astrocytes.^[^ ^33^ ^]^ However, iPSC‐derived cells have a higher potential for clinical translation and transplantation as they are more physiologically relevant to in vivo human cells,^[^ ^21^ ^]^ and their growth and differentiation may be more sensitive to specific matrix compositions.^[^ ^44^ ^]^ Therefore, we wished to investigate the capacity of CIV/FN substrates to support iPSC‐derived neuron and astrocyte outgrowth (**Figure** **1**A).

iPSC neurons grown on CIV/FN substrates displayed longer neuritis, whereas iPSC neurons grown on poly‐l‐lysine (PLL) displayed small neurites with minimal branching (Figure 1B). When quantified, the average neurite length of iPSC neurons cultured on CIV/FN was significantly enhanced with an overall increase of 2.03‐fold (Figure 1C). Sholl analysis^[^ ^45^ ^]^ was also applied to probe more detailed metrics of neuronal morphology. Maximum neurite length was significantly enhanced when neurons were grown on CIV/FN substrates compared to PLL (Figure 1D). Additionally, the sum of neurite intersections (a measure of branching and neurite length) was also significantly increased (Figure 1E). Neuronal complexity (an assessment of branching over defined distances), calculated using the Schonen ramification index,^[^ ^45^ ^]^ showed no difference, indicating that neurite extension was due to increased individual neurite length instead of neurite number (Figure 1F). Confocal microscopy and reflection imaging also revealed a significant increase in fine process length (processes ranging from 1 to 15 µm in length) indicating that CIV/FN substrates also promoted process extension in iPSC neurons at a microscale level (Figure 1G,H). Additionally, iPSC‐derived astrocytes cultured on CIV/FN substrates displayed significant differences in morphology compared to astrocytes cultured on PLL, adopting more stellate phenotypes (Figure 1I). In addition, astrocyte process extension significantly increased 2.35‐fold on CIV/FN substrates (Figure 1J) along with increased numbers of processes (Figure 1K). However, there was no difference in overall astrocyte cell area (Figure 1L), CIV/FN grown cells possessed a lower nuclear:cytoplasm ratio (a morphological measure of astrocyte reactivity^[^ ^46^ ^]^), indicating that CIV/FN substrates may promote a nonreactive, resting phenotype (Figure 1M). Analysis of astrocyte glial fibrillary acidic protein (GFAP) intensity and lactate dehydrogenase (LDH) release showed no differences across the substrates, but astrocyte metabolic activity was significantly enhanced on CIV/FN substrates at days 1 and 7 (Figure S1A–C, Supporting Information) further demonstrating CIV/FN as a growth permissive substrate. Finally, stimulated emission depletion (STED) super‐resolution microscopy revealed that qualitatively astrocyte fine process outgrowth was enhanced on CIV/FN substrates with numerous fine processes extending compared to astrocytes on PLL‐coated substrates (Figure S1D, Supporting Information).

### 2.2 Biomimetic Scaffolds Mimicking the Matrix Composition, Alignment, and Stiffness of the Spinal Cord Can Be Fabricated

**Figure 2:**
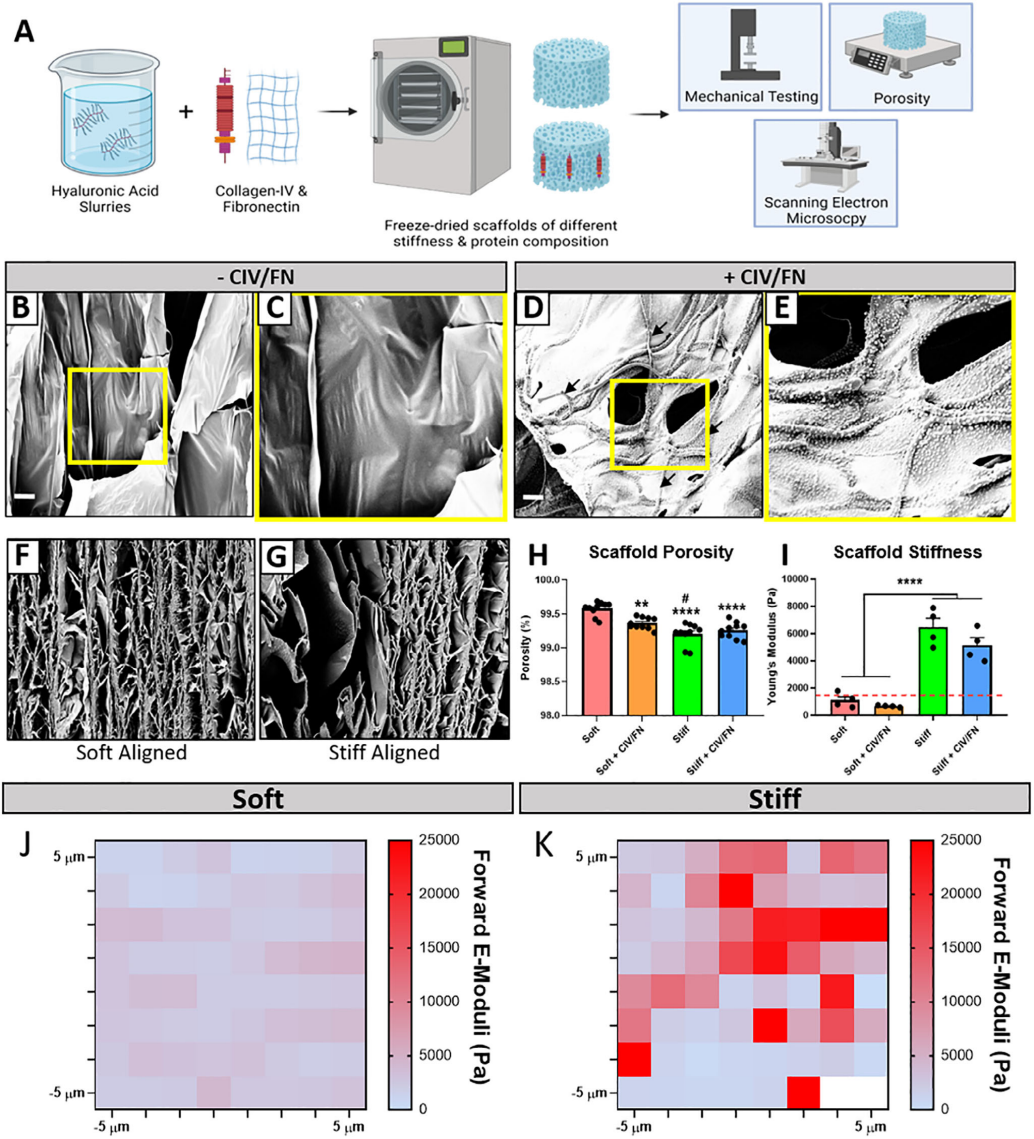
Porous biomimetic aligned spinal cord scaffolds of different stiffness and extracellular matrix composition can be manufactured. A) Freeze‐dried HyA scaffolds of different concentrations were functionalized with collagen‐IV and fibronectin (CIV/FN). B,C) Scaffolds without CIV/FN are made up of smooth HyA struts. D,E) Scaffolds functionalized with CIV/FN display HyA sheets decorated with collagen fibrils and dotted fibronectin structures. Scaffold pore architecture can be aligned in both F) soft and G) stiff scaffolds through liquid nitrogen snap freezing. H) All scaffolds are highly porous (>99%) while softer scaffolds display higher levels of porosity compared to stiffer scaffolds. I) Soft scaffolds are in line with the native stiffness of the healthy spinal cord tissue (<1.5 kPa as denoted by the red dashed line) while stiffer scaffolds exhibit supraphysiological stiffness values. J,K) Atomic force microscopy of soft and stiff scaffolds shows differences in individual scaffold strut mechanical properties. All analyses performed via one‐way ANOVA with a Tukey posthoc test.

Once CIV/FN were identified as neurotrophic proteins that could promote iPSC neuron and astrocyte growth, the matrix proteins were next incorporated into macroporous freeze‐dried HyA scaffolds, as HyA is the most abundant component of the spinal cord extracellular matrix (**Figure** **2**A). Freeze‐dried manufacturing was utilized to create highly porous scaffolds to support cell infiltration and consistent physicochemical properties with low batch‐to‐batch variability in the manufacturing process.^[^ ^47^ ^]^ Using previously developed techniques from our lab,^[^ ^33^, ^38^, ^47^ ^]^ HyA scaffolds of 3 and 10 mg mL^−1^ concentrations (soft and stiff, respectively) were functionalized with CIV/FN and snap‐frozen in polytetrafluoroethylene (PTFE) molds and underwent tailored lyophilization procedures at −40 °C to achieve unidirectional pore architectures to mimic the aligned structure of the spinal cord tissue. Scanning electron microscopy imaging revealed that CIV and FN were incorporated into the freeze‐dried HyA scaffolds with numerous microfibrillar structures decorating HyA sheets in the CIV/FN‐functionalized scaffolds (Figure 2B–E). The scaffold pore architecture contained a uniaxially aligned channel architecture in all scaffolds, and the incorporation of CIV/FN did not affect the pore alignment (Figure 2F,G). All scaffolds were also highly porous with >99% porosity, an advantageous property for cell infiltration into the scaffolds^[^ ^48^ ^]^ (Figure 2H). Mechanical testing of HyA scaffolds of different concentrations (3 and 10 mg mL^−1^) revealed that soft 3 mg mL^−1^ HyA scaffolds (soft: 1110.79 ± 265.96 Pa; soft CIV/FN: 675.49 ± 32.52 Pa) were in line with the stiffness of healthy and injured native spinal cord tissue (<1500 Pa). In contrast, supra‐physiological stiffness values (>1500 Pa) were recorded for stiffer 10 mg mL^−1^ HyA scaffolds (stiff: 6459.99 ± 672.02 Pa; stiff CIV/FN: 5119.69 ± 575.13 Pa). Moreover, scaffold CIV/FN functionalization did not significantly affect overall scaffold stiffness. Finally, atomic force microscopy showed that individual scaffold struts that are sensed at a local cellular level were softer and closer in range with the stiffness of the native spinal cord while stiffer scaffolds had significantly higher strut elastic moduli (Figure 2J,K).

### 2.3 Biomimetic Scaffolds Promote Multicellular iPSC Progenitor Spheroid Growth and Enhance Functional Capacity

**Figure 3:**
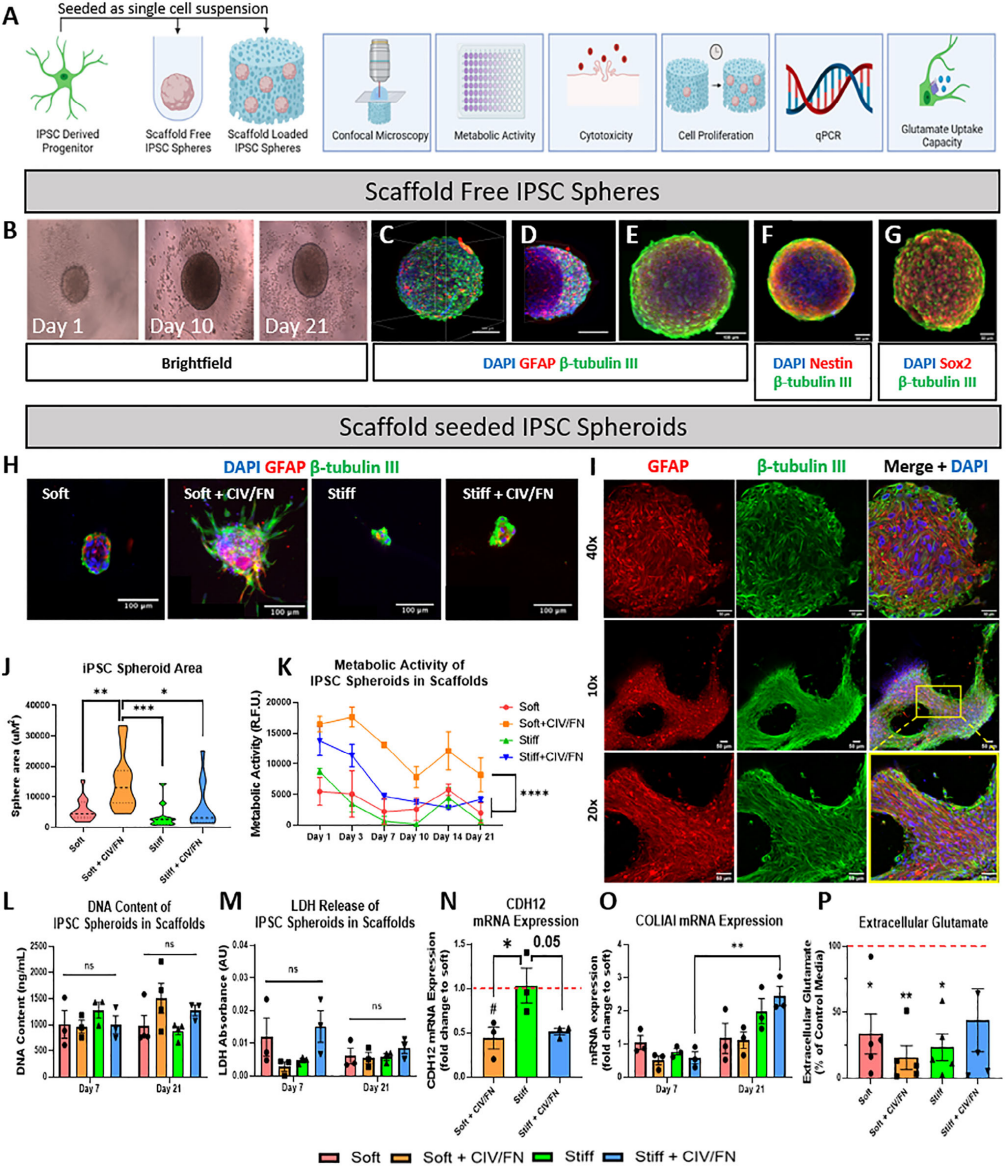
Soft collagen‐IV and fibronectin (CIV/FN) hyaluronic‐acid‐functionalized scaffolds enhance the growth, metabolic activity, scaffold colonization, and functional capacity of multicellular iPSC progenitor spheroids. A) Overview of the experimental outline. B) iPSC progenitors can form dense spheroids in scaffold‐free conditions that can survive over 21 days. C,D) Light sheet microscopy shows uniform, multicellular distribution with beta‐tubulin III positive cells (green) localized to the exterior and GFAP positive cells (red) localized to the interior of the spheroid. E) Confocal imaging shows a similar distribution of GFAP and beta‐tubulin III positive cells within spheroids. F,G) Spheroids also stain positive for Nestin and Sox2, key markers of stemness (red respectively) alongside beta‐tubulin III positive staining (green). H) Confocal imaging of iPSC progenitors showed that scaffolds could support multicellular spheroid formation. I) Confocal microscopy revealed that spheroids formed in soft + CIV/FN scaffolds were multicellular and could form dense GFAP and beta‐tubulin III positive tracts throughout the scaffold architecture (scale bar = 50 µm). J) Spheroids had a significantly larger area in soft + CIV/FN scaffolds compared to stiffer scaffolds and scaffolds without CIV/FN. K) Metabolic activity measured over 21 days was found to be significantly higher in soft + CIV/FN scaffolds compared to other scaffolds groups. L,M) All scaffolds had similar levels of DNA content and displayed similar levels of LDH release. N) Analysis of cadherin (CDH12) expression via qPCR analysis showed significantly reduced levels in iPSC progenitors seeded in soft + CIV/FN and stiff + CIV/FN scaffolds compared to soft (red‐dotted line) and/or stiff HyA only scaffold groups (*versus Stiff, #versus Soft). O) Col1a1 gene expression was also significantly enhanced over time in iPSC spheroids in stiff + CIV/FN scaffolds only. P) Extracellular glutamate uptake was significantly enhanced in soft + CIV/FN scaffold groups the most compared to baseline levels. J,N,P) Analysis via one‐way ANOVA and Tukey posthoc test. All other analyses via two‐way ANOVA, Bonferroni posthoc test. J) *N* = 3, *n* = 11–16 fields of view. K) *N* = 3.

Having successfully manufactured and characterized the biomimetic scaffolds, we next investigated whether CIV/FN‐functionalized HyA scaffolds could support the growth of trophic iPSC astrocyte progenitor cells and whether scaffold physicochemical properties influence iPSC‐derived neural cell responses (**Figure** **3**A). Several reparative cell‐based strategies have used spheroids for delivery to enhance cell survival and promote paracrine signaling,^[^ ^49^, ^50^ ^]^ and the porous nature of these scaffolds provides an interconnected soft environment that has the potential to facilitate the in situ self‐assembly of spheroids. Therefore, before investigating the ability of the 3D scaffold environment to promote spheroid formation, we first characterized the ability of iPSC astrocyte progenitors to form spheroids in scaffold‐free conditions. When grown in round‐bottom well‐plates, iPSC astrocyte progenitors successfully formed uniform spheroid structures up to 21 days^[^ ^49^ ^]^ (Figure 3B). Light‐sheet microscopy of immunofluorescently labeled spheroids showed that they formed uniform and spherical 3D structures where GFAP positive cells were found in the interior layers and beta‐tubulin III positive cells were localized to the exterior demonstrating the multipotent differentiation of the cells in these structures (Figure 3D,E). The iPSC progenitor spheroids also expressed markers of stemness such as Nestin and Sox2, indicating their suitability as immature cells ideal for delivery and integration into host tissue^[^ ^51^, ^52^ ^]^ (Figure 3F,G).

Next, we wished to examine whether the physicochemical properties of the scaffolds could be tuned to facilitate spheroid formation within the scaffolds and investigate whether scaffold stiffness and/or matrix composition could affect their formation and function. Single‐cell suspensions of iPSC astrocyte progenitors successfully formed spheroids within the porous network of all scaffold groups by day 7; however, there were clear differences in spheroid quality and size across the groups (Figure 3H). Spheroids that developed in soft CIV/FN scaffolds were significantly larger than spheroids in the stiffer and non‐CIV/FN scaffold groups, with evidence of extensive cell migration into the scaffold. In contrast, iPSC progenitors seeded in stiffer scaffolds also appeared to form spheroid‐like structures but were smaller, exhibited poorer circularity, and appeared amorphic with little or no cell migration. Spheroids were also observed to be uniform in size and distributed evenly within the scaffold structures (Figure S2A, Supporting Information). Confocal microscopy further revealed that spheroids grown within the soft CIV/FN scaffolds were multicellular, showing similar cellular organization to spheroids cultured in low‐adhesion plates (Figure 3I). Moreover, these spheroids formed dense multicellular networks throughout the scaffold with neighboring spheroids to colonize the 3D structures (Figure S2B, Supporting Information), a feature that was not observed in the other scaffold groups. Additionally, no differences in GFAP or beta‐tubulin III expression were detected across scaffold groups at day 7 or 21 following quantitative polymerase chain reaction (qPCR) analysis indicating that scaffold properties did not influence progenitor differentiation (Figure S3, Supporting Information).

Analysis of spheroid size revealed that soft CIV/FN scaffolds supported larger spheroid formation compared to the other scaffold groups (Figure 3J). Analysis of the metabolic activity of the spheroids grown in soft CIV/FN scaffolds was significantly higher compared to all other scaffold groups over 21 days of scaffold culture indicating long‐term cell survival within the scaffolds (Figure 3K). Furthermore, all scaffolds supported similar levels of DNA content (Figure 3L) without significantly exacerbating cytotoxicity (measured via LDH release) over the 21 day culture period, indicating no differences across all groups (Figure 3M). Furthermore, reverse transcription (RT‐qPCR) analysis revealed a decreased expression of mechanosignaling‐related cadherin expression (CDH12) in astrocyte progenitors grown in soft CIV/FN scaffolds while Col1a1 expression (a marker of scar‐forming behavior in astrocytes^[^ ^3^, ^29^ ^]^) was significantly increased in stiff CIV/FN scaffolds by day 21 (Figure 3N,O). Finally, scaffold properties impacted iPSC spheroid functional capacity to uptake and store glutamate (a key role of astrocytes^[^ ^53^ ^]^) with soft CIV/FN scaffolds promoting the highest levels of glutamate uptake (Figure 3P).

### 2.4 Biomimetic iPSC Progenitor‐Loaded Scaffolds Exhibit Enhanced Angiogenic Potential

**Figure 4:**
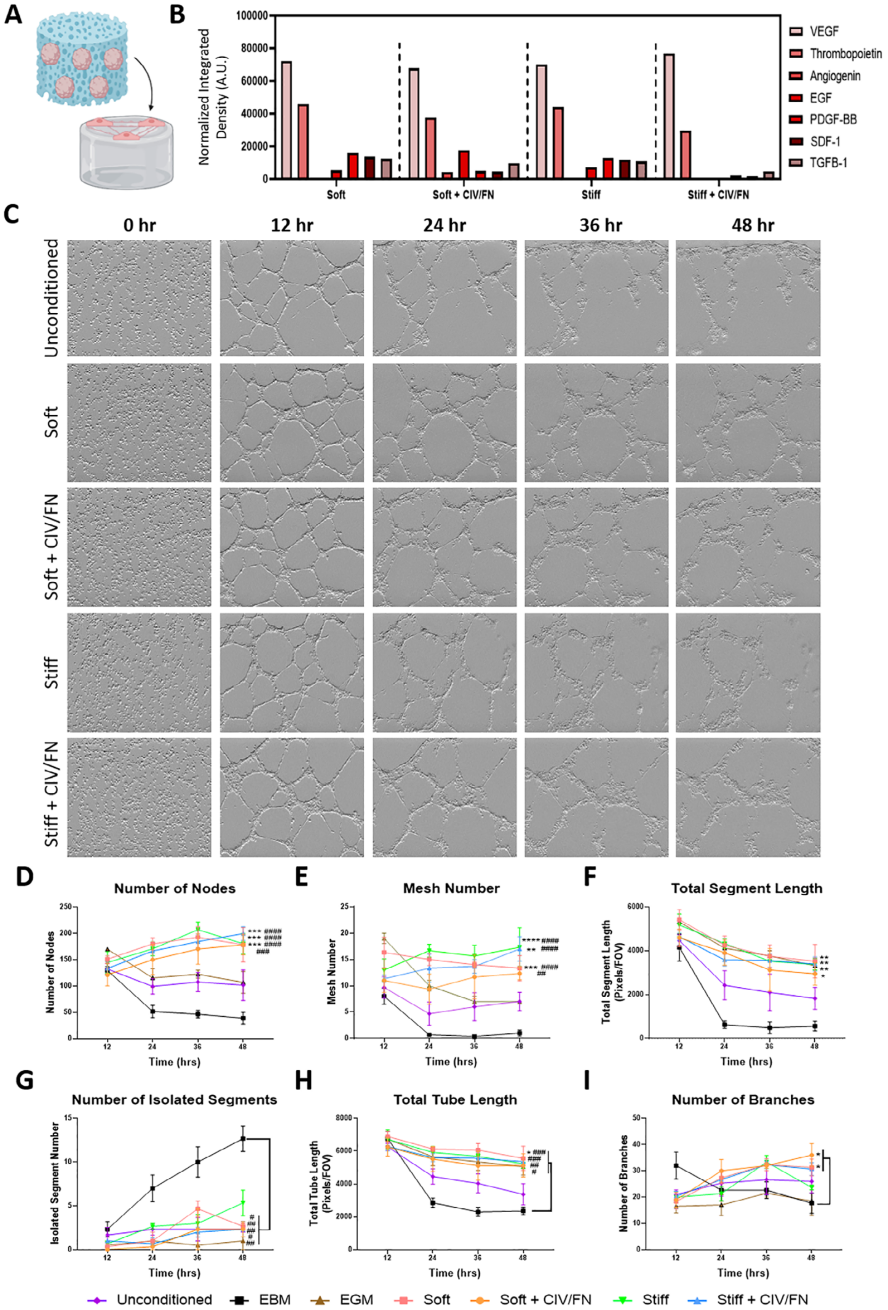
Biomimetic hyaluronic‐acid‐based scaffolds enhance the angiogenic potential of iPSC progenitors to promote the formation of vascular networks. A) Conditioned media treatments collected from iPSC progenitor‐seeded scaffolds were applied to human umbilical vein endothelial cells to determine the angiogenic potential of the scaffold‐based platforms. B) Cytokine array analysis for angiogenic factors released from iPSC‐seeded scaffolds. C) Phase‐contrast imaging revealed the effect of scaffold conditioned media treatments on endothelial tubule network formation over 48 h. Quantification of D) node number, E) mesh number (number of enclosed regions within the networks), F) total segment length (sum of tubule lengths), G) isolated segments (indicative of reduced tubule stability), H) total tube length of the vascular network, I) number of branches. D–I) Analysis via two‐way ANOVA, Bonferroni posthoc test. *versus unconditioned, #versus EBM negative control. *N* = 3 for all data.

As the local microvasculature of the spinal cord is severely damaged following injury, it was important to determine if the scaffold‐seeded iPSC astrocyte progenitors could promote the formation of vascular‐like structures (**Figure** **4**A). Cytokine release analysis revealed that scaffold‐seeded iPSC astrocyte progenitors released a similar panel of angiogenic factors, with vascular endothelial growth factor (VEGF) and thrombopoietin showing the largest and the second‐largest amount of release, respectively, across all groups (Figure 4B; Figure S1, Supporting Information). Lower levels of other key angiogenic cytokines were also detected across the different scaffold groups, with angiogenin being only detected in soft CIV/FN scaffold groups.

Conditioned media treatments collected from the different iPSC astrocyte progenitor‐seeded scaffolds were then applied to human endothelial cells to assess the effect of paracrine factors on vascular network formation over 48 h using live imaging of a tube‐forming assay^[^ ^54^ ^]^ (Figure 4C). All scaffold groups were capable of enhancing vascular‐like network formation and reduced network degradation compared to untreated, endothelial basal media (EBM, negative control group) and angiogenic cytokine‐supplemented endothelial growth media (EGM, positive control group). Image analysis revealed that the numbers of nodes (branch sites) (Figure 4D) and meshes (fully closed loops in the network) (Figure 4E) were significantly enhanced by all iPSC progenitor‐seeded scaffold media treatments indicating the induction of endothelial cells into vascular‐like structures. The total segment length was also enhanced (Figure 4F) while the number of isolated segments remained low following scaffold media treatments, indicating minimal degradation of the long tubule structures over time (Figure 4G). The total tube length was also significantly enhanced following conditioning from all scaffold groups (Figure 4H). Finally, soft and soft CIV/FN scaffold groups increased the number of branches in the vascular‐like networks, indicating that iPSC astrocyte progenitors in softer scaffolds can promote the formation of more complex vascular networks (Figure 4I).

### 2.5 Biomimetic Scaffolds Promote the Immunomodulatory Capacity of iPSC Progenitors to Influence Healthy and Injured Astrocyte Behavior

**Figure 5:**
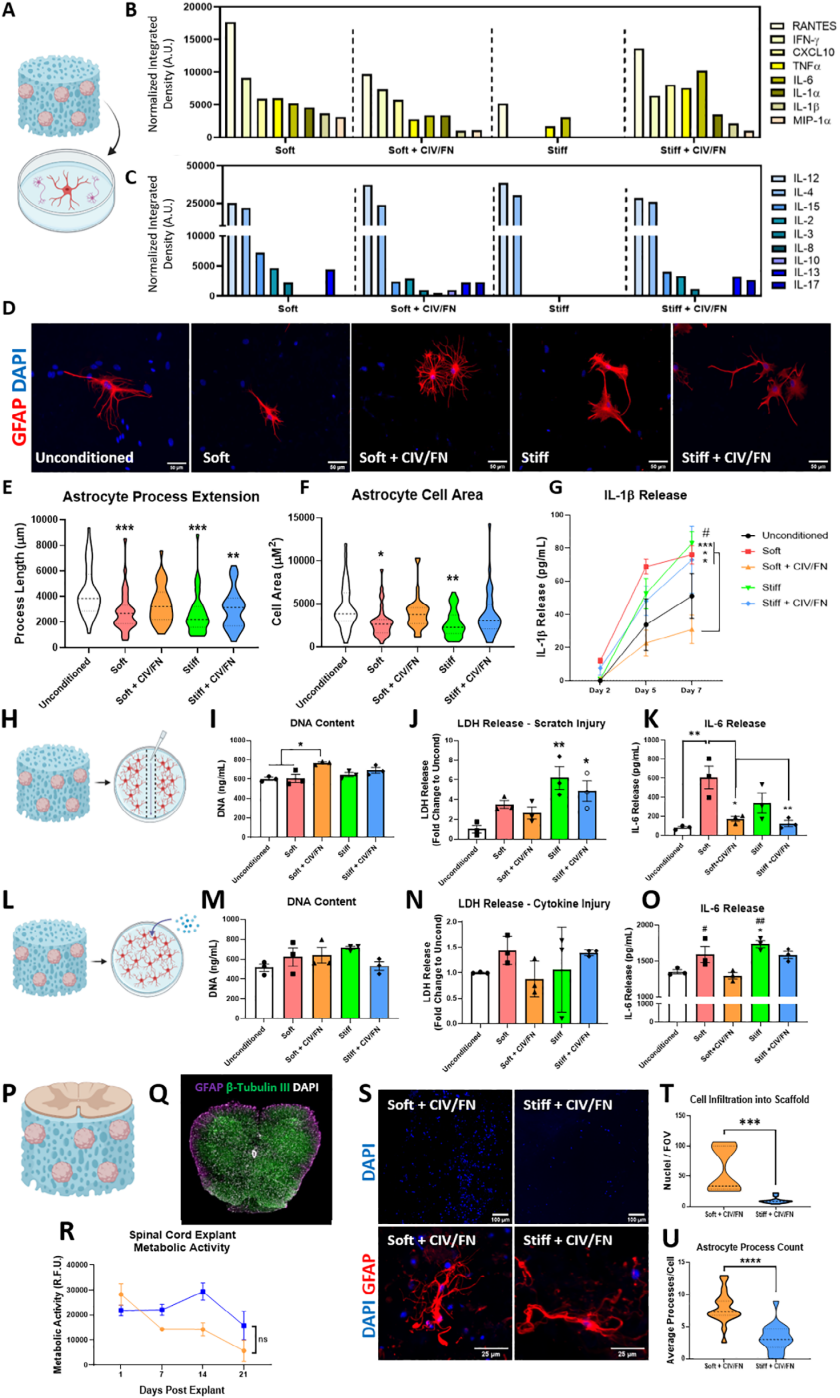
Soft collagen‐IV and fibronectin (CIV/FN) scaffolds enhance the immunomodulatory capacity of iPSC‐derived progenitors for spinal cord repair applications. A) Experimental outline of how scaffold‐conditioned treatments were applied to mixed spinal cord cultures. Cytokine array analysis reveals B) semiquantitative differences in pro‐inflammatory factors and C) immunomodulatory cytokines released from scaffold‐seeded iPSC progenitors. D) Conditioned media taken from different iPSC‐seeded scaffolds and applied to spinal cord cultures elicit different astrocyte morphologies. E,F) Quantification of astrocyte process extension and cell area following scaffold conditioning treatment. G) IL‐1β release from spinal cord cultures was significantly lower when treated by soft CIV/FN scaffolds seeded with iPSC astrocyte progenitors compared to all other scaffold media treatments. H) Experimental outline of the scratch injury performed on a monolayer of spinal cord astrocytes before applying conditioning treatments from iPSC progenitor‐seeded scaffolds. Analysis of scratch injured spinal cord astrocyte I) DNA content, J) LDH release, and K) IL‐6 release following exposure to iPSC progenitor scaffold conditioned media. L) Experimental outline of the cytokine injury performed on a monolayer of spinal cord astrocytes before applying conditioning treatments from iPSC progenitor‐seeded scaffolds. Analysis of scratch injured spinal cord astrocyte M) DNA content, N) LDH release, and O) IL‐6 release following exposure to iPSC progenitor scaffold conditioned media. P) Adult mouse spinal cord explants were cultured on iPSC‐progenitor‐seeded scaffolds. Q) Spinal cord explants could be dissected and R) cultured on iPSC‐seeded scaffolds with no significant differences in metabolic activity across 21 days (red line = soft CIV/FN scaffold, blue line = stiff CIV/FN scaffold). S,T) Cell infiltration was significantly enhanced in soft CIV/FN scaffolds compared to stiff CIV/FN scaffolds. S,U) Astrocytes that infiltrated from spinal cord explants into soft CIV/FN scaffolds displayed enhanced process counts compared to astrocytes that infiltrated into stiff CIV/FN scaffolds. D) Scale bars = 50 µm. E,F) *N* = 3, *n* = 34–39 fields of view. Analysis via one‐way ANOVA and Tukey posthoc test. G) *N* = 3. Analysis via two‐way ANOVA, Bonferroni posthoc test. I–K,M–O) *N* = 3. Analysis via one‐way ANOVA and Tukey posthoc test. R) Analysis via two‐way ANOVA, Bonferroni posthoc test. S) Scale bars = 100 µm (top) and 25 µm (bottom). T) *N* = 3, *n* = 9–16 fields of view. U) *N* = 3, *n* = 18–19 fields of view. Analysis via unpaired two‐tailed *t*‐test.

Once it was observed that iPSC astrocyte progenitor‐seeded scaffolds could elicit an angiogenic effect to support vascular network formation; it was next investigated whether the different scaffold environments could modulate inflammatory signaling from healthy and injured astrocytes (**Figure** **5**A). Analysis of the cytokine release profile of different iPSC astrocyte progenitor‐seeded scaffolds revealed that soft CIV/FN scaffolds promoted the release of a more diverse panel of anti‐inflammatory and immunomodulatory‐related cytokines compared to all other groups (Figure 5B,C). Interestingly, while present in small amounts, the anti‐inflammatory cytokine interleukin 10 (IL‐10) was only detected from iPSC progenitor spheroids in soft CIV/FN scaffolds. In contrast, soft scaffolds without CIV/FN and stiff CIV/FN scaffold groups promoted the release of higher levels of pro‐inflammatory cytokines compared to soft CIV/FN scaffolds.

As scaffold physicochemical properties were observed to influence the inflammatory release profile of the seeded cell cargo, the effect of different iPSC progenitor‐seeded scaffolds to elicit gliotrophic responses in primary mouse spinal cord astrocytes, cells that would initially encapsulate an implant, was assessed. Scaffold‐conditioned media treatments promoted qualitatively different astrocyte morphologies (Figure 5D). Softer scaffold media treatments encouraged astrocytes with small cell soma containing several branches while media collected from stiffer scaffolds promoted larger, hypertrophic astrocyte morphologies with less branching. Morphological analysis revealed that astrocyte process extension was reduced significantly when treated with media from stiffer and CIV/FN‐free scaffold groups while soft CIV/FN scaffold groups showed no overall difference compared to unconditioned control levels (Figure 5E). Similarly, astrocyte cell area was significantly decreased compared to control levels when treated by CIV/FN‐free HyA scaffolds (Figure 5F). No differences were observed the nuclear:cytoplasm ratios^[^ ^33^ ^]^ and GFAP intensity^[^ ^55^ ^]^ (Figure S1, Supporting Information), morphometrics commonly used to assess astrocyte reactivity. Interestingly, iPSC progenitors seeded in soft CIV/FN scaffolds lowered IL‐1β (a cytokine that is chronically elevated post‐SCI^[^ ^56^ ^]^) release compared to unconditioned controls and all other scaffold conditioned media treatments, indicative of a possible anti‐inflammatory effect (Figure 5G). Contrastingly, media from stiffer and CIV/FN‐free iPSC progenitor‐seeded scaffold groups increased pro‐inflammatory IL‐1β release.

Next, we wished to determine if scaffolds of different physicochemical properties could impact iPSC progenitor gliotrophic capacity to influence astrocyte responses to physical and cytokine injury (mimicking the primary and secondary responses to SCI respectively).^[^ ^35^, ^57^ ^]^ Human primary spinal cord astrocytes underwent a scratch injury before being treated with iPSC progenitor‐seeded scaffold conditioned media for 48 h (Figure 5H). Scratch‐assay wound closure area was not significantly impacted by scaffold conditioning (Figure S3, Supporting Information). However, injured astrocyte DNA content was significantly higher in soft CIV/FN scaffold‐treated groups compared to unconditioned controls and soft HyA only scaffold‐treated groups indicating a higher number of astrocytes present following the injury and scaffold conditioned media exposure (Figure 5I). Astrocytes that received media from unconditioned and soft CIV/FN scaffold groups showed similar levels of cytotoxic LDH release, while media from stiff and stiff CIV/FN scaffold groups significantly exacerbated LDH release (Figure 5J). Analysis of pro‐inflammatory IL‐6 release from astrocytes was also low in control, soft CIV/FN, and stiff CIV/FN scaffold conditioned groups but was significantly increased in astrocytes treated with soft HyA only scaffold media (Figure 5K). To replicate the inflammatory conditions seen in the secondary phase of SCI, astrocytes underwent an established cytokine injury through exposure to inflammatory cytokines IL‐1α, tumor necrosis factor alpha (TNFα), and component 1q (C1q)^[^ ^8^, ^57^ ^]^ before being treated similarly with scaffold conditioned media (Figure 5L). In contrast to scratch‐injured astrocytes, cytokine‐injured astrocytes showed no difference in DNA content, LDH release, or metabolic activity (Figure 5M,N; Figure S3, Supporting Information). However, similar to the scratch injury, astrocytes that received media from soft CIV/FN scaffolds showed lower levels of IL‐6 release while astrocytes treated with soft‐only and stiff‐only scaffold media significantly increased IL‐6 release (Figure 5O).

To test whether the biomimetic iPSC progenitor‐seeded scaffolds could promote reparative processes when directly in contact with complex injured neural tissues, more clinically relevant aged adult mouse spinal cord explants that demonstrate a poor intrinsic capacity to regrow were utilized (Figure 5P). Spinal cord explants were first assessed for long‐term survival in serum‐free media and demonstrated no reductions in viability compared to serum‐supplemented media over 21 days (Figure 5Q; Figure S4, Supporting Information). Next explants were cultured for 21 days on both soft CIV/FN and stiff CIV/FN scaffolds seeded with iPSC astrocyte progenitors and exhibited stable metabolic activity in both groups (Figure 5R). Cellular infiltration from spinal cord explants into the scaffolds assessed using 4',6‐diamidino‐2‐phenylindole DAPI staining of cryosections was significantly enhanced in the soft CIV/FN scaffolds seeded with iPSC astrocyte progenitors compared to the stiffer scaffolds after 21 days, indicating a more supportive environment for host spinal cord tissue interaction (Figure 5S,T). When spinal cord astrocytes migrated into softer scaffolds, they displayed a significantly higher process count and less hypertrophic morphologies compared to stiffer scaffolds (Figure 5U) demonstrating a stronger capacity to support adult spinal cord explant astrocytes and enhance cellular infiltration in softer scaffold environments.

### 2.6 Biomimetic Scaffolds Enhance the Neurotrophic Capacity of iPSC Progenitors to Promote Growing, Mature, and Injured Neuronal Growth

**Figure 6:**
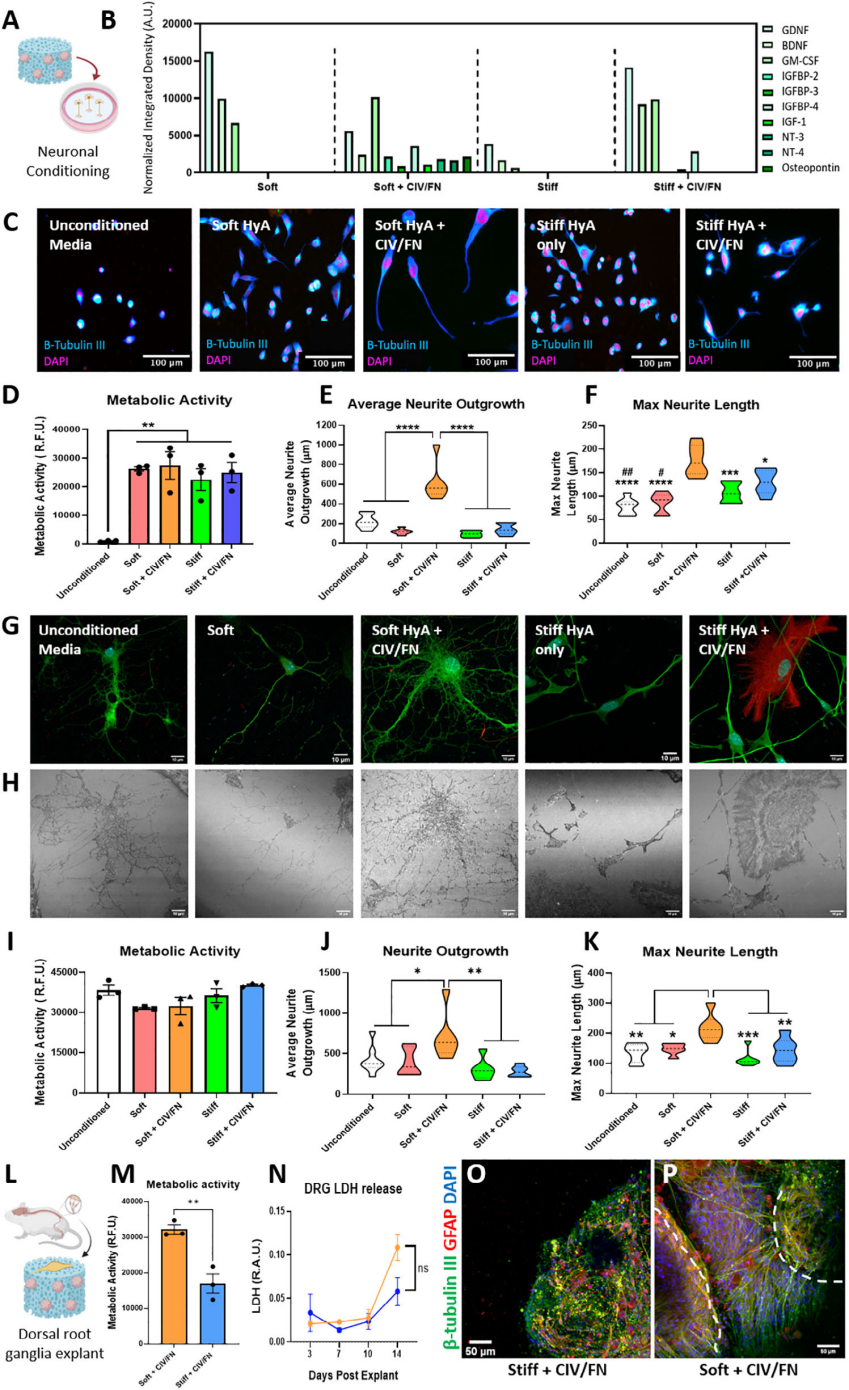
Soft collagen‐IV and fibronectin (CIV/FN) scaffolds enhance the neurotrophic capacity of iPSC astrocyte progenitors. A) Experimental outline of iPSC progenitor‐seeded scaffold conditioning of neurons. B) Cytokine array analysis for neurotrophic factors released from iPSC‐progenitor‐seeded scaffolds. C) Representative images of NSC‐34 neurons that were cultured in unconditioned and iPSC progenitor‐seeded scaffold‐conditioned media over 7 days. D) Metabolic activity was significantly increased in neurons that received conditioned treatments from iPSC progenitor‐seeded scaffold groups. E,F) Average and max neurite outgrowth of NSC‐34 neurons exposed to soft CIV/FN iPSC progenitor scaffold conditioned media was significantly increased compared to all other groups. Additionally, neurons conditioned by stiff CIV/FN scaffolds showed significant increases in max neurite length compared to unconditioned controls (##*p* < 0.01) and soft only (#*p* < 0.05) groups. G,H) Representative confocal and reflection images of spinal cord neurons exposed to iPSC progenitor‐seeded scaffold conditioned media show differences in neurite density. I) Cell metabolic activity was not affected by any conditioning treatments compared to control levels. J,K) Spinal cord neurons treated with soft CIV/FN media displayed significant increases in average and max neurite lengths compared to all other groups. L) Adult mouse dorsal root ganglia (DRG) explants were cultured on soft CIV/FN and stiff CIV/FN iPSC progenitor‐seeded scaffolds. M) DRGs cultured on soft iPSC progenitor‐seeded scaffolds showed higher metabolic activity compared to stiffer scaffold groups at day 14. N) Scaffold stiffness did not significantly affect LDH release across 14 days of culture. O) DRGs cultured on stiff scaffolds did not extend neurites. P) DRGs cultured in softer scaffolds (bottom left dashed line) displayed enhanced neurite extension, connecting with iPSC progenitor spheroids (top right dashed line). B) Scale bars = 100 µm. C) *N* = 3. D,E) *N* = 3, *n* = 36–54 fields of view. Analysis via one‐way ANOVA and Tukey posthoc test. F,G) Scale bars = 10 µm. H–J) *N* = 3, *n* = 6–9 coverslips averaging values across multiple fields of view. Analysis via one‐way ANOVA and Tukey posthoc test. L) *N* = 3. Analysis via unpaired, two‐tailed *t*‐test. M) *N* = 3. Analysis via two‐way ANOVA, Bonferroni posthoc test. N,O) Scale bar = 50 µm.

Once the angiogenic and immunomodulatory capacity of the iPSC astrocyte progenitor‐seeded scaffolds had been studied, we next wished to determine whether scaffold physicochemical properties could promote the neurotrophic capacity of iPSC astrocyte progenitors to enhance the extension of growing, mature, and injured neurons (**Figure** **6**A). First, analysis of neurotrophic cytokines released from iPSC astrocyte progenitor‐seeded scaffolds revealed that soft CIV/FN scaffolds promoted the release of numerous different growth‐inducing cytokines from iPSC progenitors compared to all other scaffold groups (Figure 6B). In contrast, the other scaffold groups promoted the release of fewer cytokines, but with brain‐derived neurotrophic factor (BDNF) and glial‐derived neurotrophic factor (GDNF) being released in higher amounts.

To test the neurotrophic capacity of the iPSC‐seeded scaffolds to promote the growth of growing motor neurons, conditioned scaffold media were applied to cultures of growing NSC‐34 neurons (Figure 6C). Distinct qualitative morphological differences were observed where neurons exposed to soft CIV/FN scaffold conditioning displayed long neurites. Contrastingly, unconditioned control groups and all other scaffold conditioned groups did not extend neurites, with cells displaying rounded morphologies. Analysis of neuronal metabolic activity showed a significant increase in all groups after 1 week of scaffold media treatments compared to controls (Figure 6D). However, only soft CIV/FN scaffolds seeded with iPSC astrocyte progenitors increased neurite extension compared to all other groups with a 3.4‐fold increase in average neurite outgrowth compared to control‐treated neurons (Figure 6E; Figure S5, Supporting Information). Similarly, the max neurite length was also enhanced when neurons were exposed to media from soft CIV/FN scaffolds seeded with iPSC astrocyte progenitors compared to control and other scaffold media (Figure 6F).

Next, to determine whether iPSC progenitor‐seeded scaffolds could enhance neurite outgrowth in mature neurons, similar scaffold conditioning was applied to mouse primary spinal cord cultures. Soft CIV/FN scaffold‐conditioned media enhanced the density of fine neurites that extended from the cell body while neurons treated with other scaffold media displayed fewer process extensions (Figure 6G). Reflection imaging further revealed a denser coverage in neurons exposed to soft CIV/FN scaffold conditioning (Figure 6H). The metabolic activity of the spinal cord cultures was not affected following scaffold conditioning (Figure 6I). However, the average neurite outgrowth (Figure 6J) and max neurite length (Figure 6K) were enhanced following exposure to soft CIV/FN scaffolds seeded with iPSC astrocyte progenitor media, indicating a potent effect to enhance neuronal outgrowth.

Finally, to test whether the developed scaffold platforms could successfully promote axonal regrowth when placed directly in contact with more complex neural tissue, injured adult DRGs were cultured on iPSC astrocyte progenitor‐seeded scaffolds (Figure 6L). After 14 days of DRG culture, metabolic activity was observed to be significantly higher in co‐cultures of soft CIV/FN scaffolds compared to stiffer CIV/FN scaffolds, and no differences in LDH release were detected (Figure 6M,N). In the soft CIV/FN scaffolds only, DRGs and iPSC progenitor spheroids extended numerous beta‐tubulin III positive neurites toward each other, indicating that soft, but not stiffer, CIV/FN‐functionalized scaffolds can facilitate connection with aged, injured neural tissue explants (Figure 6O,P).

## 3 Discussion

The objective of this study was to investigate the ability of a biomimetic scaffold implant to enhance the angiogenic, immunomodulatory, and neurotrophic capacity of seeded iPSC astrocyte progenitors for spinal cord repair applications in a stiffness and matrix‐dependent manner. Native spinal cord matrix proteins CIV/FN elicited a potent neurotrophic effect, driving an over 2‐fold increase in iPSC neurite outgrowth. When CIV/FN was incorporated into biomimetic scaffolds matching the stiffness of the spinal cord, soft CIV/FN‐functionalized HyA scaffolds successfully acted as delivery vehicles to support the growth of trophic patient‐derived iPSC astrocyte progenitors.^[^ ^18^, ^58^ ^]^ Soft CIV/FN scaffolds promoted the formation of large multicellular spheroids that over time could extend throughout the scaffold producing dense GFAP and beta‐tubulin III positive processes, indicating the capacity of the scaffold to support complex interconnected iPSC neural cell populations. Furthermore, soft CIV/FN scaffolds were able to enhance the angiogenic potential of the spheroids to promote vascular network formation, attenuate the inflammatory responses of healthy and injured astrocytes, and promote neurite extension of growing mature and injured neurons. Finally, when DRG and spinal cord explants were cultured directly on the iPSC astrocyte progenitor‐seeded scaffolds, only soft scaffolds enhanced cellular infiltration, astrocyte process extension, and directed axonal outgrowth between DRG and iPSC progenitor‐spheroids, indicating a stiffness‐dependent neurotrophic effect of the biomimetic scaffolds to enhance the trophic potential of the seeded cell cargo.

Stem and progenitor cell transplantations via injection into the inflammatory environs of the injured spinal cord typically result in low retention and viability.^[^ ^13^, ^14^, ^24^ ^]^ We proposed that seeding clinically relevant patient‐derived trophic cells into freeze‐dried scaffolds that can be reproducibly manufactured with low batch‐to‐batch variability^[^ ^47^, ^48^ ^]^ would be advantageous for site‐specific delivery to the injury site. Additionally, while scaffolds can shield the cell cargoes from the surrounding inflammatory environment, we observed that tuning scaffold physicochemical properties could simultaneously enhance the reparative effectiveness of the cells that colonized the scaffold.^[^ ^14^, ^24^ ^]^ We show that soft CIV/FN scaffolds mimicking the physicochemical properties of the healthy spinal cord successfully promoted the formation of large iPSC astrocyte progenitor spheroids within the porous scaffold architecture. Simultaneously, the soft CIV/FN scaffolds enhanced cellular viability, trophic cytokine production, and glutamate uptake functional capacity of the trophic iPSC astrocyte progenitors in a stiffness‐dependent manner. Previous research has shown that freeze‐dried scaffolds can enhance the survival of single‐cell suspensions of embryonic neural stem cell grafts in mouse models of a spinal cord crush injury by offering site‐specific delivery to the injury site.^[^ ^24^ ^]^ However, the formation of spheroids within freeze‐dried scaffolds demonstrated here is not reported and is demonstrably advantageous for promoting the paracrine release of trophic cytokines and aiding cell survival through enhanced cell–cell signaling.^[^ ^59^ ^]^ In particular, the ability of the soft CIV/FN scaffolds to support the development of multicellular tracts from the spheroids may indicate that these scaffolds can support the infiltration and growth of dense regrowing axonal tracts^[^ ^60^ ^]^ through the macroporous scaffold and across the lesion site overtime following implantation. These soft scaffolds also enhanced the survival of seeded cells (as demonstrated by the enhanced viability observed across 21 days compared to all other scaffold groups) which remains a significant challenge for the effectiveness of patient derived cell‐based therapies for spinal cord repair.^[^ ^13^, ^14^ ^]^ Upon analyzing the cytokine release profile in more detail, it was observed that a broad spectrum of cytokines were released from the scaffold‐seeded iPSC astrocyte progenitor spheroids, where scaffold stiffness and matrix composition drastically influenced the diversity of cytokines released.

Scaffold‐seeded iPSC astrocyte progenitors released several known pro‐angiogenic factors and demonstrated an enhanced capacity to support vascular network formation. Following spinal cord injury, the innate angiogenic processes triggered by the host tissue to respond to the initial trauma are frequently inadequate to distribute sufficient blood supply and improve axonal recovery.^[^ ^61^ ^]^ Here we demonstrate that the developed scaffold platforms can support the release of a panel of angiogenic cytokines including vascular endothelial growth factor^[^ ^62^ ^]^ and angiogenin,^[^ ^63^ ^]^ which are critical for promoting the formation and stabilization of new blood vessels. The findings from our tube‐forming assay align with previous studies as a positive indicator for the angiogenic potential of the iPSC astrocyte progenitor‐seeded scaffolds for neural tissue repair. Increases in the number of mesh networks, nodes, branches, and length of branches in tube‐forming assays have been shown by others to correlate with increased vascular structures which was associated with enhanced motor recovery in rats following SCI.^[^ ^64^ ^]^ Furthermore, the diversity of angiogenic cytokines released from the iPSC astrocyte progenitor‐seeded scaffolds is likely capable of supporting the different phases of angiogenesis. For example, studies have shown that VEGF plays an important role in the initiation of angiogenesis^[^ ^62^, ^65^ ^]^ while transforming growth factor (TGF‐β) is primarily involved in vessel stabilization.^[^ ^66^ ^]^ Revascularization in the spinal cord remains a challenging but an important facet of therapeutic strategies to not only limit ischemic damage but to encourage neuronal regrowth along re‐vascularized tissue.^[^ ^67^ ^]^ Many cell‐based therapies utilizing neurons for delivery may not be able to promote vascular repair due to the fact that the lack of angiogenic cytokines neurons can produce and release compared to astrocytes.^[^ ^68^, ^69^ ^]^ The use of iPSC astrocyte progenitors within this study also offers an attractive alternative approach to promote vascular repair by delivering cells capable of merging with endogenous astrocytes of the blood–brain spinal barrier that can release angiogenic cytokines to promote functional recovery following injury.^[^ ^61^, ^70^, ^71^ ^]^

The mechanical properties of the scaffold environment significantly influenced the release of neurotrophic and immunomodulatory cytokines from the formed iPSC astrocyte progenitor spheroids, where soft CIV/FN scaffolds promoted a more diverse cytokine release profile compared to stiffer and CIV/FN‐free HyA scaffolds. Our group and others have previously shown that material stiffness can influence cytokine release from astrocytes^[^ ^27^ ^]^ and that neural cell behavior can be influenced through interactions with their surroundings more generally.^[^ ^43^ ^]^ However, while the effect of substrate and environmental stiffness on neural progenitor cell differentiation has been well reported, the effect of stiffness on the trophic and immunomodulatory capacity of implantable cells is less well studied.^[^ ^43^ ^]^ Here we report that softer biomimetic scaffolds enhanced the release of a diverse panel of neurotrophic cytokines in a stiffness‐dependent manner and that scaffold physicochemical properties more generally affect the neurotrophic profile of implanted cells. Similarly, soft CIV/FN scaffolds demonstrated enhanced glutamate uptake, indicating that iPSC astrocyte progenitor functionality^[^ ^72^ ^]^ can also be influenced by scaffold physicochemical properties. These findings have implications for the role of iPSC astrocyte progenitors reducing glutamate excitotoxicity at the injury site when implanted.^[^ ^22^, ^73^ ^]^ While previous work from our group has used these biomimetic scaffolds to look at physiochemical interactions with cells of the surrounding environment,^[^ ^33^ ^]^ this study presents new insights on the potent effect of scaffold stiffness and matrix composition on trophic‐seeded cell cargo from human patient cell sources for delivery. We believe these insights can help guide strategies for iPSC delivery for neural applications where the number of human clinical trials using iPSC‐derived cells continues to grow.^[^ ^74^ ^]^

By optimizing scaffold stiffness and matrix composition to mimic the native spinal cord tissue, soft CIV/FN scaffolds enhanced the paracrine activity of iPSC astrocyte progenitors to encourage neurite outgrowth. Only soft CIV/FN scaffolds seeded with iPSC astrocyte progenitors significantly promoted increases in neurite outgrowth of up to 3.4‐folds from growing motor neurons and mouse primary spinal cord neurons. Previous studies have shown that freeze‐dried scaffolds can improve the viability of seeded neural stem cells for SCI repair in mice, but not the neurotrophic ability of the seeded cells to enhance axonal growth.^[^ ^24^ ^]^ Our findings demonstrate that scaffold design complimenting the physicochemical properties of the healthy cord can promote diversity in the cytokine secretome of seeded iPSC astrocyte progenitors while also simultaneously enhancing cell viability and functional capacity. The secretome of transplanted stem and progenitor cells is broad (containing cytokines, lipoproteins, and extracellular vesicles),^[^ ^68^, ^75^, ^76^, ^77^, ^78^ ^]^ and cytokine production is known to be modulated in response to a range of stimuli, including physicochemical cues such as changes in environmental stiffness^[^ ^27^, ^43^ ^]^ and matrix composition.^[^ ^35^ ^]^ Several studies have highlighted the importance of material stiffness in neural cell culture,^[^ ^27^, ^79^, ^80^ ^]^ but typically over broader supra‐physiological stiffness ranges, outside the range of the spinal cord (i.e., high kPa–MPa range).^[^ ^27^, ^81^, ^82^ ^]^ Few studies have recapitulated a 3D scaffold environment similar to the native cord tissue (i.e., in the range of Pa)^[^ ^37^, ^83^ ^]^ and tested how this affects the cytokine release profile of implantable cell cargo. Therefore, these data highlight the effectiveness of utilizing biomimetic scaffold platforms to enhance the potential of iPSC astrocyte progenitor cell cargo to promote significant neurite outgrowth from both motor neurons and primary spinal cord neurons. Moreover, we show that small changes in the mechanical properties of 3D scaffold environments (in the range of 1000s of pascals) can have significant effects on the trophic potential of the delivered cell cargo. The effect of changes in stiffness within this relative Pascal range is of importance as it aligns with the changes observed in injured spinal cord tissue, where lesion cores within the injury site are observed to stiffen but still remain in the low kPA range.^[^ ^84^ ^]^

Additionally, the work conducted here builds on previous findings from our group and provides novel insights into the use of scaffolds with stiffer physicochemical properties for spinal cord repair applications. Previously, we have demonstrated that stiffer scaffold environments limit the growth of uninjured growing cell line neurons and astrocytes.^[^ ^33^ ^]^ Here, scaffolds manufactured with supra‐physiological stiffness altered iPSC astrocyte progenitor responses so that their glio‐trophic and immunomodulatory potential was limited and instead appeared to exacerbate astrocyte inflammatory responses and cytotoxicity in models of primary and/or secondary cord injury.^[^ ^25^ ^]^ Contrastingly, soft CIV/FN scaffolds promoted lower levels of inflammatory cytokine release, and did not limit astrocyte growth or promote inflammation or cytotoxicity in any instance. To date, the primary metric of assessing a scaffold's success in promoting spinal cord repair typically focuses on promoting axonal outgrowth instead of assessing how a scaffold also influences glial cell responses.^[^ ^24^, ^33^ ^]^ We provide additional rationale for the biomimetic design of scaffolds, which not only enhances positive neurotrophic responses from seeded patient‐derived cell cargo to promote neurite outgrowth but also attenuates inflammation and influences the immunomodulatory responses of patient‐derived cells.

The soft CIV/FN scaffolds seeded with iPSC astrocyte progenitors promoted robust growth and enhanced cellular responses in two neural tissue explants, demonstrating the effectiveness of the biomimetic scaffold platform to promote growth and infiltration from different aged multicellular neural tissues. These scaffolds promoted enhanced astrocyte infiltration from spinal cord explants into the scaffolds and promoted significantly higher amounts of astrocyte processes, indicative of the adoption of nonreactive phenotypes by infiltrating glial cells. These findings demonstrate the effectiveness of our developed scaffold slice culture approach for studying spinal cord tissue interactions with scaffolds and underline the importance of optimizing scaffold stiffness to support host tissue astrocyte migration and survival.^[^ ^37^, ^85^ ^]^ The influence of scaffold properties to promote astrocyte migration from spinal cord explants into the 3D structures is important to both prevent adverse scarring around the scaffold surface following implantation and to encourage and support the growth of axons into the scaffold along astrocyte networks.^[^ ^3^, ^18^, ^22^ ^]^ As demonstrated here, the soft CIV/FN scaffolds may promote astrocyte colonization of the scaffold without eliciting scarring responses^[^ ^6^, ^86^ ^]^ following implantation to the injured cord environment. The cytokine profile of iPSC astrocyte progenitors within these scaffolds also showed an increased anti‐inflammatory cytokine IL‐10 release, linking scaffold properties to astrocyte anti‐inflammatory activity in line with previous findings.^[^ ^33^ ^]^

These biomimetic iPSC astrocyte progenitor‐seeded scaffolds also enhanced the extension of re‐growing injured axons between dorsal root ganglia and progenitor spheroids within soft scaffolds, indicating the impact of scaffold stiffness in facilitating implantable cell and host neural tissue interactions within 3D environments. Previous studies have demonstrated that environmental stiffness^[^ ^82^, ^87^, ^88^ ^]^ and the paracrine activity of transplanted cells^[^ ^18^, ^22^ ^]^ can influence axonal regrowth individually. Additionally, previous work from our group has shown that softer scaffolds can promote neurite extension and infiltration from dorsal root ganglia compared to stiffer scaffold environments. Here, we show that physicochemically optimized scaffolds which deliver support cell delivery can promote the physical interaction between locally delivered spheroids and injured axons within scaffold environments driving axonal regrowth, an important goal of SCI repair strategies.^[^ ^2^, ^14^ ^]^ Further testing of the iPSC progenitor‐seeded scaffolds in hemi‐ and/or full transection in vivo models of SCI may provide more information on the survival and function of these cells post implantation. However, future studies should seek to use mouse iPSC‐derived cells for use in rodent models of SCI as the delivery of human iPSC‐derived cells in vivo, while more clinically relevant, requires the use of immune‐suppressed rodents, which may not display physiologically relevant neural repair processes. Overall, the positive responses observed in aged mouse cord explants that have a poor intrinsic capacity to regrow,^[^ ^33^ ^]^ in a 3D environment, demonstrate the reparative potential of this iPSC astrocyte progenitor‐seeded scaffold platform and how tuning scaffold properties can enhance the effectiveness of cell therapy strategies more broadly. These findings have important implications for enhancing scaffold‐based strategies for treating chronic injuries among an increasingly ageing demographic of patients living with SCI.^[^ ^89^, ^90^ ^]^

In the future, there are areas that may be of interest for further investigation. While we demonstrate that N‐cadherin and Col1a1 gene expression, which are involved in astrocyte mechanosignaling and scar formation, respectively,^[^ ^29^ ^]^ are affected by scaffold properties within this study, the exact mechanisms driving changes in iPSC astrocyte progenitor behavior remain unknown. Further analysis via RNA sequencing may offer further information into the mechanisms underlying these changes and provide additional information into how cell delivery strategies may be enhanced through tuning scaffold physicochemical properties.

Expanded analysis of the secretome of iPSC astrocyte progenitors within this study through more widespread proteomics may also offer information on additional neurotrophic, immunomodulatory, and angiogenic cytokines as well as vesicles that may be released from the different seeded scaffolds within this study.^[^ ^68^ ^]^ Finally, due to the complex nature of spinal cord injury, advanced multifaceted therapeutic strategies combining scaffold‐based cell delivery with drug delivery,^[^ ^13^ ^]^ gene delivery,^[^ ^92^ ^]^ and/or electroactive materials to support electrostimulation^[^ ^92^, ^93^ ^]^ may show promise for promoting functional repair of injured spinal cord tissue.

## 4 Conclusion

This study demonstrated that tuning the physicochemical properties of implantable scaffolds that mimic the healthy spinal cord has a pronounced beneficial effect on the angiogenic, immunomodulatory, and neurotrophic potential of trophic patient derived iPSC astrocyte progenitors. This soft collagen‐IV and fibronectin‐functionalized hyaluronic acid scaffold provides an ideal platform to not only deliver clinically relevant stem‐cell‐based therapeutics but to enhance their neurotrophic effectiveness through tuning of scaffold stiffness and matrix composition.

## 5 Experimental Section

All reagents were purchased from Sigma–Aldrich (Ireland) unless stated otherwise. Wash steps refer to 3× Dulbecco's phosphate‐buffered saline (dPBS) washed for 5 min at room temperature (RT) unless stated otherwise. All cells were mycoplasma tested using a mycoplasma detection kit (Invivogen, USA).

### IPSC‐Derived Neuronal and Astrocyte Culture on Extracellular Matrix Substrates

iPSC‐derived neural and astrocyte progenitor cells were differentiated from the NAS2 iPSC line purchased from the European bank for iPSCs according to previously established protocols.^[^ ^21^, ^94^ ^]^ Differentiated iPSC‐derived neurons (>30 days old) were cultured in neuronal maintenance media (NMM: consisting of Dulbecco's modified eagle medium (DMEM) F12 and neurobasal media (Gibco, UK) (50:50), 1% penicillin/streptomycin (P/S), 1% glutamax, 1% MEM nonessential amino acids (NEAA), 0.2% N21 (Biotechne, UK), 1% B27, 50 µm 2‐mercaptoethanol (Thermo Fisher Scientific, UK)). iPSC‐derived astrocytes (>60 days) were cultured in “Serio EL” media^[^ ^95^ ^]^ consisting of advanced DMEM F12 (Gibco, UK), 1% P/S, 1% glutamax, 1% MEM NEAA, 1% N21 (Bio‐Techne, UK), 0.2% B27, 20 ng mL^−1^ human epidermal growth factor (EGF) and 20 ng mL^−1^ human leukemia inhibitory factor (LIF) (both from PeproTech, UK) until confluent. The cells were passaged by detaching with Accutase, centrifuged (1000 rpm, 3 min) and resuspended in NMM or Serio EL media and plated onto Celltrex (1:100, R&D Systems, UK) coated 6‐well plates for expansion. For cell morphology studies, both neurons and astrocytes were seeded directly onto PLL or CIV/FN‐coated coverslips (1 × 10^4^ cells per coverslip, 10 µg mL^−1^ concentrated coatings) as described previously^[^ ^96^ ^]^ and cultured for 7 days. iPSC neuron culture media were supplemented with 50 µm ROCK inhibitor Y‐27632 dihydrochloride (Axon Medchem, Netherlands) to aid cell survival post‐seeding. Cells were then fixed in 4% paraformaldehyde (PFA) (20 min, RT).

### Immunohistochemical Characterization of iPSC Morphology

iPSC‐derived neurons and astrocytes were immunostained for beta‐tubulin III (T3952, Sigma–Aldrich) and GFAP (556327, BD Pharmigen, UK) using goat antirabbit 555 secondary antibodies (Invitrogen, UK) in combination with Phallodin‐488, respectively. Cells imaged and analyzed as described in previous studies^[^ ^45^ ^]^ to assess change in cell outgrowth or by using a Sholl analysis^[^ ^45^ ^]^ ImageJ macro to determine the effect of CIV/FN on cell morphology. From these data, the Schonen ramification index,^[^ ^45^ ^]^ a measure of neuronal complexity, was calculated via maximum intersections across the Sholl circles divided by the primary neurite count. Finally, iPSC‐derived neurons were also imaged using confocal microscopy and reflection imaging (Stellaris 8‐STED, Leica, Germany) to analyze the fine process extension of actin filaments (as highlighted by overlapping reflection images and positive Phalloidin‐488 staining). Fine process length was measured manually using the ImageJ tracer tool and an average value per field of view was acquired. Alternatively, for STED staining of iPSC astrocyte actin structure, cells were stained with Alexa Fluor 594 Phalloidin (1:1000, Invitrogen, UK) as described in a previous study.^[^ ^34^ ^]^ For STED microscopy, images were acquired utilizing a Leica STELLARIS 8 τ‐STED 3D Falcon microscope using a 93×/1.30 glycerin immersion lens using laser settings described previously.^[^ ^34^ ^]^

### Scaffold Manufacture and Characterization

Hyaluronic acid scaffolds of 3 and 10 mg mL^−1^ (referred to as soft and stiff, respectively) with and without CIV/FN were manufactured as previously described.^[^ ^33^, ^47^, ^92^ ^]^ Briefly HyA sodium salt (1.6–1.8 MDa) and dH_2_O were added together to produce HyA solutions of 10 or 3 mg mL^−1^ and were mixed for 24 h. Next, 1‐ethyl‐3‐(3‐dimethylaminopropyl) carbodiimide (EDAC) (0.8 g/100 mL solution) and adipic acid dihydrazide (9.16 g/100 mL solution) were added in excess and allowed to mix for 24 h. 1 m hydrochloric acid was added to lower the HyA slurry pH to 4, initiating the crosslinking reaction (4 °C, for 18 h). The pH was then raised to 7.4 using 1 m sodium hydroxide and the crosslinked Hya was dialyzed for 6 days in sequential solutions of sodium chloride (NaCl) solution (29.5 g L^−1^ dH_2_O, 24 h, RT); fresh NaCl solution (29.5 g L^−1^ dH_2_O, 24 h, RT); 20% ethanol (24 h, RT); dH_2_O replaced every 24 h (72 h, RT). Collagen‐IV and fibronectin were then added to the HyA solution (0.1 mg mL^−1^ each), syringe triturated (× 30 times) and then degassed to a pressure of 4000 mTorr (Leybold D16B, Biopharma, UK). To produce an individual scaffold, 250 µL of each slurry solution was pipetted into custom‐designed PTFE molds coupled to metal baseplate using previously described protocols.^[47]^ To form longitudinally aligned pores, the filled moulds were first placed in liquid nitrogen (5 min) before being loaded into a freeze dryer (VirTis Advantage Pro, Biopharma, UK) set at −40 °C for 25 h. Once completed the formed scaffolds were rehydrated in an ethanol series of 100% ethanol (1 h, RT); 90% ethanol (1 h, RT), and 70% ethanol (overnight, 4 °C) followed by incubation in 14 mm EDAC and 5.5 mm *N*‐hydroxysuccinate (NHS) in 70% ethanol (2 h, RT) to crosslink collagen‐IV/fibronectin into the HyA before performing a final PBS wash.

### Characterization of Scaffold Properties

Uniaxial mechanical testing was conducted on scaffolds immersed in a PBS bath using a mechanical tester equipped with a 5 N load cell (Z050, Zwick Roell, Germany). Strain rates of 10% min^−1^ were applied until failure and the stress calculated from the applied force to the scaffold surface area and the strain calculated from scaffold displacement in relation to height between 10% and 20% before the compressive modulus was calculated from the slope of the stress/strain curve. To study the porous microarchitecture, scaffolds were analyzed via scanning electron microscopy. Scaffolds were sectioned longitudinally and transversely using a tissue chopper (McElwain, USA), fixed to adhesive carbon stubs (Tedpella, USA). Dry samples were then sputter coated with a 5 nm layer of an 80:20 mixture of gold and palladium using a Cressington 108 auto sputter coater. Imaging was carried out using a Zeiss Ultra field emission scanning electron microscopy using the InLens detector at an accelerating voltage of 5 kV, an aperture size of 30 µm, and a working distance of 5 mm, with images acquired at varying magnifications on both the longitudinal and cross‐sectional surface. Scaffold porosity was measured via gravimetry.^[^ ^97^ ^]^ Scaffold height, diameter (mm), and weight (mg) were measured using a micrometer and scales, respectively. The following formula was then used to calculate porosity

(1) $$\mathrm{Porosity}=1-P_{\mathrm{scaffold}}/P_{\mathrm{material}}$$

where *P*_scaffold_ = mass/volume and *P*_material_ = 1.8 ± 0.1 g cm^−3^ (density of HyA).

For nanoindentation measurements, scaffolds were embedded in optimal cutting temperature compound without fixation. The samples were then snap‐frozen and cryo‐sectioned into 100 µm thick slices. The sections were washed in PBS and placed on glass slides and held in place with plastic holders. Samples were mounted on an atomic force microscope (CoreAFM, Nanosurf, Switzerland) and immersed in PBS before being subjected to tissue stiffness measurements. A gold‐coated silicon nitride cantilever with a 10 µm borosilicate glass spherical tip and a 0.1 N m^−1^ Force Constant (CP‐qp‐CONT‐BSG‐B, NanoandMore GMBH, UK) was used. Indentation was carried out in an 8 × 8 array across a 10 µm × 10 µm area. Samples were assumed to be incompressible, and a Poisson's ratio of 0.5 was used. Ana software (Nanosurf) for automated force mapping was used to calculate the Young's modulus of each sample using a fit of the Hertz model.

### Analysis of iPSC Astrocyte Progenitor Spheroid Formation

Cell spheroids in scaffold‐free conditions were first generated and analyzed before determining whether biomimetic scaffolds could support spheroid formation and whether scaffold properties influenced spheroid morphology. iPSC astrocyte progenitors were seeded at 1 × 10^4^ cells per well as single‐cell suspensions into round‐bottom low adhesion 96‐well plates in Serio EL media (Nunc Sphera plates, Thermo Fisher, UK). iPSC astrocyte progenitors were then centrifuged to pellet the cells (1 min, 1000 rpm) before incubating and feeding every 1–2 days by removing and replacing 50% of the media. Spheroid growth and morphology were recorded over 21 days using an inverted microscope (DMIL model, Leica, Switzerland) before they were fixed in 4% paraformaldehyde (30 min, RT), washed (PBS), and immunostained for GFAP, beta‐tubulin III, Sox2 (MAB2018, Biotechne, UK) and Nestin (MAB2736, Biotechne, UK) as described previously.^[^ ^47^, ^96^ ^]^ Briefly, spheroids were incubated in 1% bovine serum albumin (BSA)–PBS (1 h, RT) before incubating in primary antibody solution (diluted 1:500 in 0.3% Triton‐X—1% BSA–PBS) overnight at 4 °C. Spheroids were washed in PBS and incubated in secondary antibody solution (diluted 1:500 in 0.3% Triton‐X–1% BSA–PBS) for 2 h at RT before being incubated in DAPI solution (1:500 in PBS) for 30 min, RT, and undergoing a final PBS wash. For light‐sheet fluorescence microscopy, imaging was performed using a light‐sheet Z.1 microscope (Zeiss, Germany) as described in previous work.^[^ ^96^ ^]^ For confocal imaging, spheroids were embedded in 0.5% low‐melting agarose by using a wide‐bore 1 mL syringe. Imaging was then performed as described in previous work^[^ ^96^ ^]^ using an Examiner.Z1 confocal microscope (Zeiss, Germany).

For scaffold‐based experiments, iPSC astrocyte progenitors were seeded onto scaffolds in a single‐cell suspension at 2.5 × 10^5^ cells per scaffold in Serio EL media. The media (50%) were replaced every 24 h and the conditioned media collected and stored at −20 °C until later used for LDH analysis, enzyme‐linked immunosorbent assays (ELISAs), glutamate uptake analysis or use in conditioning experiments to assess the angiogenic, immunomodulatory or neurotrophic effects of released factors in the conditioned media. Alamar blue analysis was performed on days 3, 7, 14, and 21 according to manufacturer's guidelines at 300 µL per well, 1 h at 37 °C. On days 7 and 21, scaffolds were then processed for one of the following: I) Picogreen assay (Invitrogen, UK) to measure DNA content according to the manufacturer's recommended protocols; II) fixed in 4% PFA for 30 min at RT before undergoing immunostaining and imaging to study cell morphology; III) dissociated and lysed in Qiazol lysis solution (3 scaffolds/500 µL of Qiazol) in RNA‐free eppendorfs before storing at −80 °C for qPCR analysis. For high‐resolution imaging, beta‐tubulin III, GFAP, Phallodin‐488, and DAPI immunostained scaffolds were imaged using a Zeiss Examiner.Z1 confocal microscope (Zeiss, Germany) at either 10× or 20× magnification before post‐processing 3–4 representative *z*‐stack images of spheroid images into maximum projections. Spheroid area and distribution within the scaffolds were analyzed from thresholded images using ImageJ.

### IPSC Astrocyte Progenitor Gene and Protein Analysis

ELISA (Biotechne, UK) and Picogreen (Invitrogen, UK) analysis of iPSC astrocyte progenitor‐seeded scaffolds were performed according to manufacturer's instructions. For qPCR isolation and analysis, the cell lysate was collected and transferred to a −80 °C freezer for 48 h. The samples were thawed, and the scaffold‐free lysate was transferred to RNase‐free eppendorfs. RNA isolation, reverse transcription, and qPCR analyses were then performed as described in previous studies. Collagen‐I (Col1a1: Hs_COL11A1_1_SG, ID: QT00088711, Qiagen, UK) and cadherin (CDH12: Hs_CDH12_1_SG, ID: QT00018963, Qiagen, UK) expressions were then analyzed. Results were calculated using the ΔΔ*CT* method in Microsoft Excel. Media collected from iPSC progenitor‐loaded scaffolds on days 3, 7, 14, and 21 were analyzed for LDH)release using an LDH CyQuant assay kit (Invitrogen, UK) according to the manufacturer's instructions. Analysis of astrocyte‐mediated glutamate uptake^[^ ^7^ ^]^ was performed on collected conditioned media using a fluorometric assay according to the manufacturer's instructions (Abcam, UK) and uptake was calculated relative to unconditioned control media. Cytokine array analysis was carried out on collected conditioned media using an 80‐target cytokine array membrane (Abcam, UK) according to the manufacturer's instructions and the acquired densitometry measurements were analyzed using ImageJ.

### Assessing the Angiogenic Potential of iPSC Astrocyte Progenitor‐Seeded Scaffolds

A human umbilical vein endothelial cell (HUVEC) tube formation assay was performed to assess the angiogenic potential of iPSC astrocyte progenitor‐seeded scaffolds. HUVECs (C2519A, Lonza, Switzerland) were resuspended in either 200 µL of iPSC astrocyte progenitor‐seeded scaffold conditioned or respective control unconditioned media (20 000 cells per well), in triplicate, in 96‐well plates containing Geltrex LDEV‐Free Reduced Growth Factor Basement Membrane Matrix (Biosciences, Ireland). EGM2 media supplemented with accompanying media kit endothelial cell growth factors (C‐22010, Promo Cell, Germany) were used as a positive control for this experiment, while endothelial basal media without supplementation (Lonza, Switzerland) were used as a negative control. Live cell imaging was carried out using a Zeiss Cell discoverer 7 automated imaging system at a rate of 1 image per hour over 48 h. Analysis of tube formation was carried out using the Angiogenesis analyzer plugin for ImageJ.

### Assessing Immunomodulatory Capacity of iPSC Astrocyte Progenitor‐Seeded Scaffolds

To investigate the immunomodulatory capacity of released factors from iPSC astrocyte progenitor‐seeded scaffolds, conditioned media were applied to resting and injured astrocytes before assessing changes in astrocyte morphology, reactivity, cytotoxicity, and/or pro‐inflammatory response. GFAP‐immunostained astrocytes in the mixed spinal cord cultures isolated from C57BL/6 wild‐type (WT) mice aged 3–9 days old were imaged and analyzed using ImageJ to assess GFAP intensity, nuclear:cytoplasm ratio, process extension, and cell area to determine differences in response to each of the conditioning treatments. To determine whether iPSC astrocyte progenitor‐seeded scaffolds release trophic factors that are capable of changing astrocyte behavior post injury where they become reactive, two injury assays were performed: 1) a scratch assay to mimic the initial “primary” mechanical injury^[^ ^35^ ^]^ and 2) a cytokine injury, to mimic the inflammation‐induced “secondary” injury environment.^[^ ^8^, ^57^ ^]^ To implement both assays, human primary spinal cord astrocytes (1820, ScienceCell, USA) were cultured as described previously^[^ ^33^ ^]^ and seeded onto PLL‐coated 48‐well culture plates at 2 × 10^4^ cells per well and cultured for 5 days to form a confluent monolayer. A scratch assay was carried out by scoring a single line along the full diameter of the well. Alternatively, the cytokine injury assay was applied by administering IL‐1α (3 ng mL^−1^, Peprotech, UK), TNFα (30 ng mL^−1^, Cell Signaling Technology, USA), and complement C1q (MyBioSource, USA) (400 ng mL^−1^) in growth media (500 µL per well) for 24 h to induce a neurotoxic astrocyte phenotype.^[^ ^57^ ^]^ Thereafter, the media were replaced with either fresh unconditioned or conditioned media every 24 h. Scratch injuries were imaged immediately (0 h), at 24 and 48 h post‐injury to measure the scratch area over time using an inverted bright‐field culture microscope (Leica, Germany). Scratch area was then calculated from acquired images using manual tracing tool in ImageJ. For both injury types, the supernatant was collected at 24 and 48 h for LDH (Invitrogen, UK) and IL‐6 ELISA (Biotechne, UK) analysis before measuring metabolic activity via Alamar blue assays (300 µL per well, 1 h, 37 °C). Finally, cells were lysed to study proliferation at 48 h via Picogreen assay as described previously.

### Assessing the Neurotrophic Capacity of iPSC Astrocyte Progenitor‐Seeded Scaffolds

To test the neurotrophic capacity of iPSC astrocyte progenitor‐seeded scaffolds collected conditioned media was applied to different neuronal models to investigate changes in neurite length. NSC‐34 mouse motor neuron cells (CLU140, Cedarlane Labs, Canada) were seeded at 5 × 10^3^ cells on coverslips precoated with poly‐l‐lysine (10 µg mL^−1^). NSC‐34 neurons were cultured for 24 h, before adding unconditioned or conditioned media collected from iPSC astrocyte progenitor‐seeded scaffolds every 2–3 days (500 µL per well) for 6 days. Separate cultures were grown in the following media to ensure that effects due to conditioning with iPSC astrocyte progenitor‐seeded scaffold media were due to the release of paracrine factors from iPSC astrocyte progenitors: I) Serio only media to ensure the differentiation factors EGF and LIF did not impact neuronal outgrowth;^[^ ^95^ ^]^ II) conditioned media from scaffold‐free spheroids cultured in low adhesion plates; III) NSC‐34 growth media (DMEM high glucose, 10% FBS, 1% P/S, 1% l‐glutamine) and differentiation media (DMEM F12, 1% FBS, 1% P/S, 1% l‐glutamine, and 10 µm all‐trans retinoic acid) to be used as negative and positive controls for neurite outgrowth respectively; IV) conditioned media collected from cell‐free scaffolds of different physicochemical properties to ensure degraded scaffold components were not driving changes in conditioning treatments. All neurons were metabolically assayed after 7 days of growth using the Alamar blue assay (Invitrogen, UK) according to the manufacturer's instructions and thereafter processed for fixation in 4% PFA in PBS (20 min, RT). All neurons were stained/immunostained for Phalloidin‐488 and beta‐tubulin III, respectively, as outlined earlier, before being imaged and analyzed using ImageJ to determine the average and max neurite length using manual tracing of the longest neurite/field of view.

Additionally, spinal cords were extracted from C57BL/6 WT mice aged 3–9 days old. Tissue harvesting was approved by the RCSI Research Ethics Committee (REC202005013). A mixed culture of primary spinal cord cells (containing neurons, astrocytes and other resident spinal cord cells) was obtained as described previously^[^ ^93^ ^]^ and seeded at 2.5 × 10^4^ cells per well onto poly‐l‐lysine (10 µg mL^−1^) coated coverslips. Cells were cultured in neurobasal plus media, 2% B27 plus, 0.25% glutamax (all Gibco, UK), 1% P/S, and 10% heat‐inactivated horse serum for 24 h before gradually replacing the media with serum‐free neurobasal plus media. The cells were allowed to grow for 7 days before being treated with conditioned media from iPSC astrocyte progenitor‐seeded scaffolds, where conditioned media were applied every 2–3 days (500 µL per well). Unconditioned Serio EL media were also used for conditioning as a control treatment (500 µL per well). Cell supernatant was harvested every 2–3 days for LDH cytotoxicity and pro‐inflammatory IL‐1β ELISA analysis and analyzed as previously described. Alamar blue analysis was then performed on day 7 of conditioning before fixing cells in 4% paraformaldehyde, immunostained with beta‐tubulin III, imaged, and analyzed as previously described for NSC‐34 cells. Finally, immunostained neurons were imaged via reflection and confocal microscopy to qualitatively assess outgrowth following conditioning.

### Neural Tissue Explant Scaffold Culture and Analysis

To test the effect of iPSC astrocyte progenitor‐seeded scaffolds on DRG and spinal cord explants soft and stiff HyA scaffolds functionalized with CIV and FN were seeded with iPSC astrocyte progenitors for 7 days. Tissue explants were isolated form 20 weeks old, wild‐type C57BL/6 male mice kindly donated by fellow researchers from the Research Ireland‐FutureNeuro Centre in RCSI in observance of the 3Rs principles.^[^ ^98^ ^]^ Whole spinal cord and DRGs were next dissected using the hydraulic extrusion dissection method^[^ ^99^ ^]^ and then immediately placed in media (DMEM F12, 1% penicillin/streptomycin, 1% l‐glutamine, and 25% heat‐inactivated horse serum (Gibco, UK)). Immediately following extrusion, L1–L5 lumbar spinal cords were sectioned transversely into 500 µm thick sections using a tissue chopper (McElwain, USA) and placed directly on iPSC astrocyte progenitor‐seeded scaffolds in Serio EL media. Similarly, isolated DRGs were placed directly on scaffolds in Serio EL media. Tissue explants were fed with Serio EL every 48 h and allowed to survive for 21 days. Alamar blue assay was carried out on days 1, 7, 14, and 21 to assess explant health and collected supernatant from DRG‐mounted scaffolds analyzed for cytotoxic LDH release as described previously. All explant scaffold cultures were fixed at day 21 in 4% PFA (30 min, RT). Spinal cord explants were cryoprotected using a 30% sucrose solution and cryosectioned into 50 µm thick sections and mounted on Superfrost glass slides (Thermo Fisher Scientific, Ireland) before being immunostained for DAPI and GFAP (both 1:500 dilution). Sectioned samples were imaged using confocal microscopy to study cellular infiltration and astrocyte morphology. Cellular morphology and astrocyte process count were measured manually using ImageJ.

### Statistical Analysis

Statistical analysis was carried out using GraphPad Prism 8.0. All data were first subjected to a Shapiro–Wilk's normality test before applying either parametric or nonparametric analysis. Where two treatments were compared an unpaired, two‐tailed *t*‐test was used. Where more than one treatment was compared a one‐way ANOVA with a Tukey posthoc test was carried out. Where more than one treatment was compared across two factors, a two‐way analysis of variance (ANOVA) with Bonferroni posthoc test was carried out. All experiments were performed in triplicate and were compromised of multiple technical replicates. Results were expressed as mean ± standard error of the mean (SEM) in all instances. In all instances * = *p* < 0.05, ** = *p* < 0.01, *** = *p* < 0.001, and **** = *p* < 0.0001.

## Conflict of Interest

The authors declare no conflict of interest.

## Supporting information



Supporting Information

## Data Availability

The data that support the findings of this study are available from the corresponding author upon reasonable request.
