# Supplementary material for: Biomimetic Scaffolds Enhance iPSC Astrocyte Progenitor Angiogenic, Immunomodulatory, and Neurotrophic Capacity in a Stiffness and Matrix‐Dependent Manner for Spinal Cord Repair Applications
Source: Adv Healthc Mater. 2025 May 19;14(16):2500830. doi: 10.1002/adhm.202500830 (PMC12184084; doi:10.1002/adhm.202500830)
Supplement: Supplementary file 1 — Supporting Information [file ADHM-14-0-s001.docx]

**Supporting Information**

**Biomimetic scaffolds enhance iPSC astrocyte progenitor angiogenic, immunomodulatory and neurotrophic capacity in a stiffness and matrix-dependent manner for spinal cord repair applications**

**Cian O’Connor^1,2,3^**, Ian Woods^1,2,3^, Sarah F. McComish^4,5^, Sean Kerr^1^, Matthew McGrath^1,2^, Juan Carlos Palomeque^1,2^, Jack Maughan^1,2,6^, Tara McGuire^1,2^, Maeve A. Caldwell^4,5^, Adrian Dervan^1,2,3^, & Fergal J. O’Brien*****^1,2,3^

^1^Tissue Engineering Research Group, Department of Anatomy & Regenerative Medicine, Royal College of Surgeons in Ireland (RCSI), Dublin, Ireland

^2^Advanced Materials and Bioengineering Research Centre (AMBER), RCSI & TCD, Dublin, Ireland

^3^Trinity Centre for Biomedical Engineering, Trinity College Dublin (TCD), Dublin, Ireland

^4^Department of Physiology, TCD, Dublin, Ireland

^5^Trinity College Institute of Neuroscience, TCD, Dublin, Ireland

^6^School of Physics, TCD, Dublin, Ireland


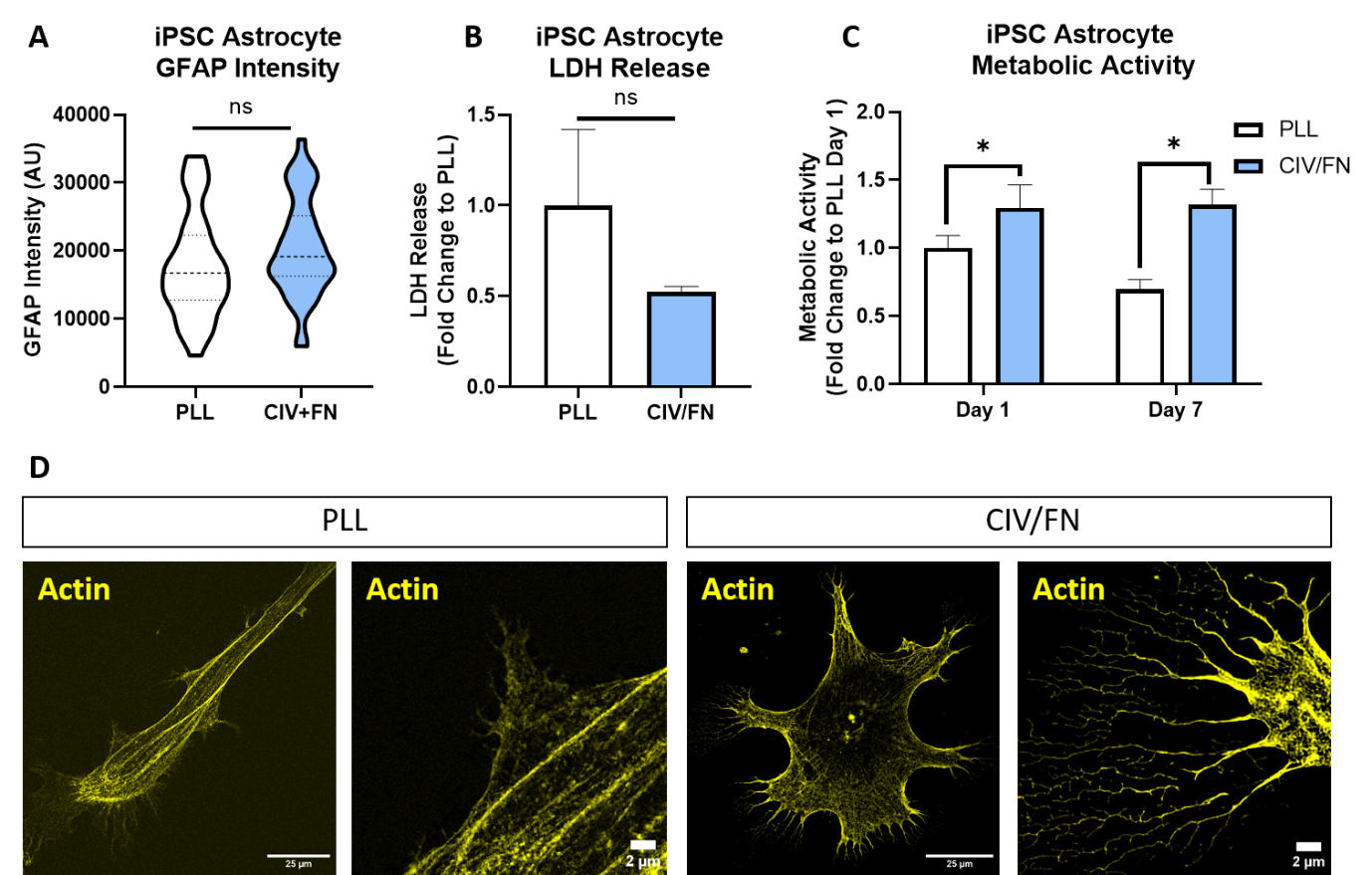


**Figure S1. Collagen IV and fibronectin substrates significantly enhanced iPSC-derived neuronal and astrocyte outgrowth (expanded analysis)**. A) IPSC astrocyte GFAP intensity is not affected by CIV/FN. B) LDH release from iPSC astrocytes on CIV/FN substrates displayed a trending, but non-significant decrease compared to astrocytes cultured on PLL. C) Metabolic activity of iPSC astrocytes cultured on CIV/FN substrates was significantly higher compared to astrocytes on PLL at both days 1 & 7. D) STED imaging shows nanoscale differences in astrocyte actin extension where fine actin protrusions can be observed extending from iPSC astrocytes on CIV/FN substrates. A) N=3, 36-38 cells. B-C) N=3. A-B) Analysis via unpaired two-tailed t-test. C) Analysis via two-way ANOVA with a Bonferroni post hoc test.


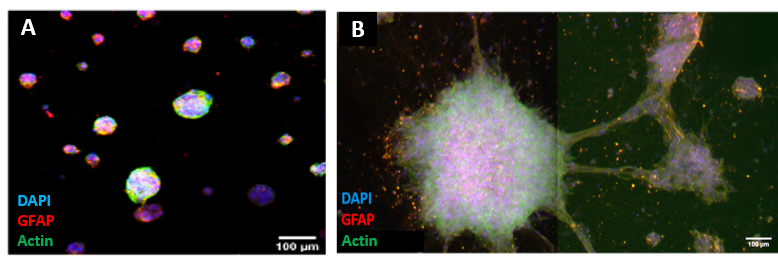


**Figure S2. Distribution and lower magnification images of iPSC astrocyte progenitor scaffold seeded spheroids**. A) Confocal image showing uniform spheroid formation and even distribution throughout the scaffold architecture. B) Lower magnification image of iPSC astrocyte progenitor spheroids connecting in soft CIV/FN scaffolds. Scale bars = 100 µm.


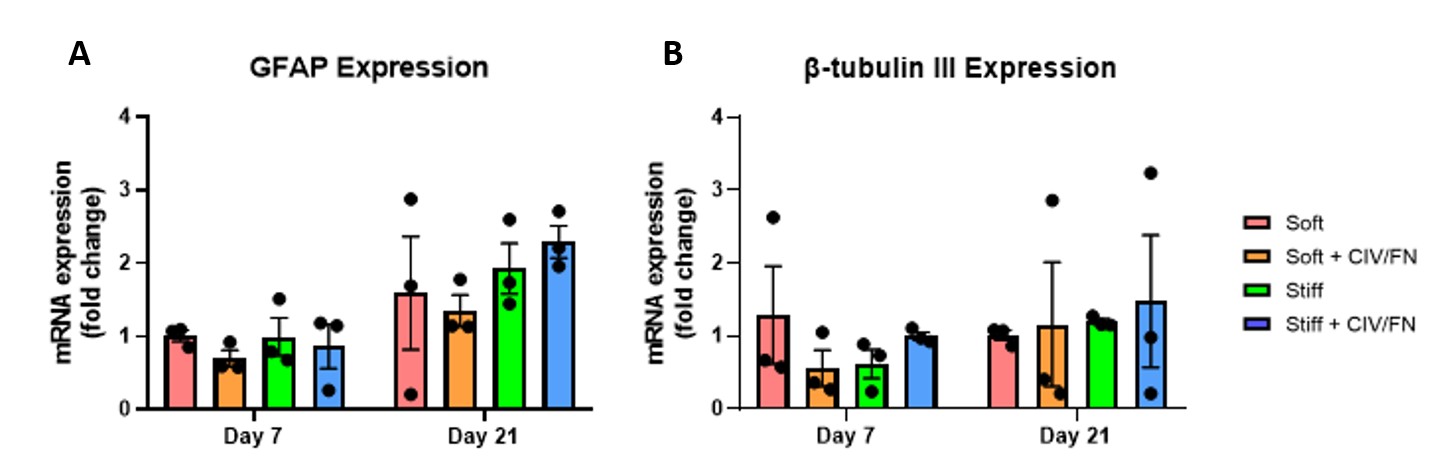


**Figure S3. Scaffold properties do not affect iPSC astrocyte progenitor differentiation**. Analysis of iPSC astrocyte progenitor (A) GFAP and (B) Beta-tubulin III gene expression across different scaffold groups shows no effect of scaffold stiffness or matrix composition on cell differentiation markers. Analysis via two-way ANOVA, Bonferroni post hoc test. N=3.


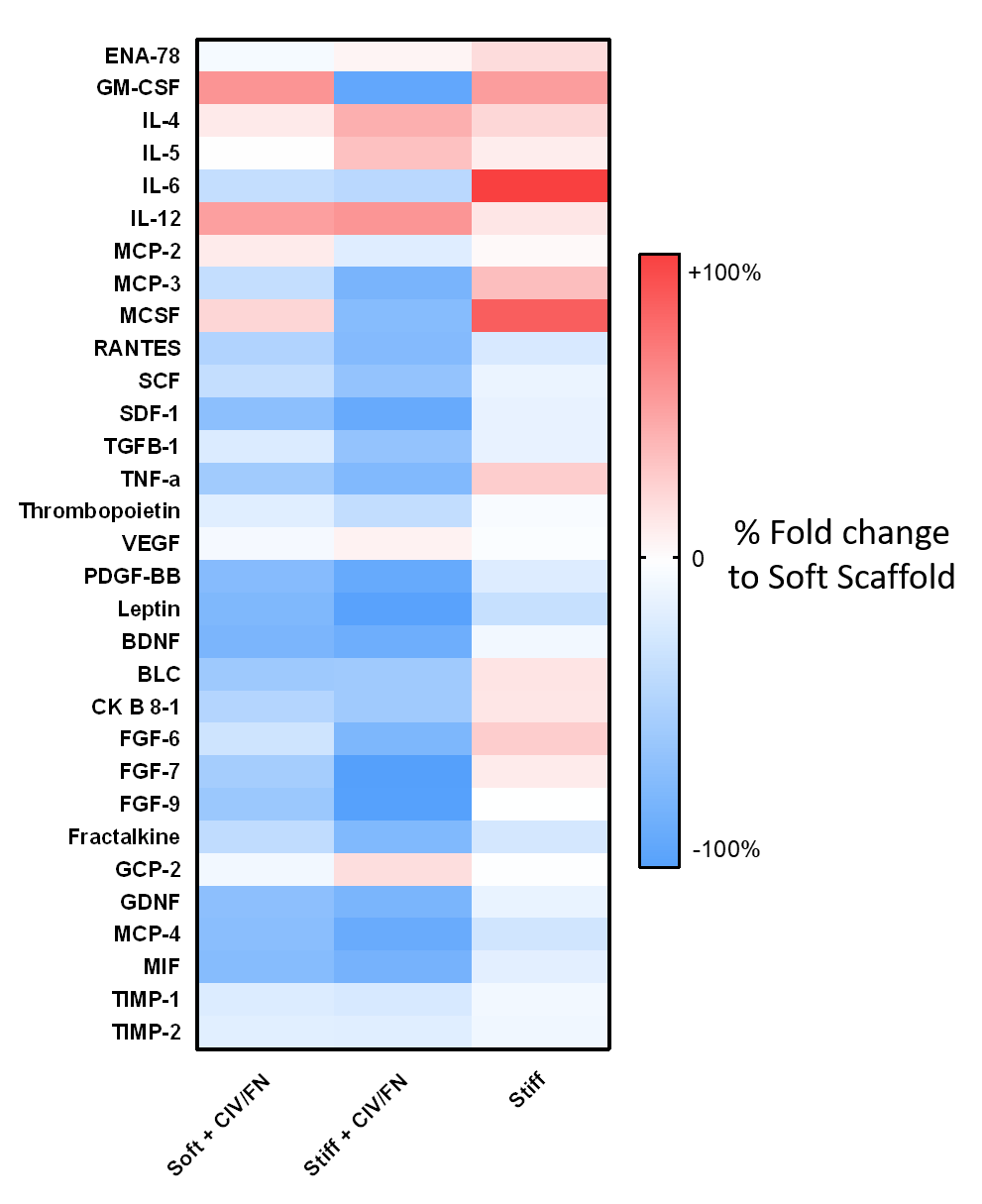


**Figure S4. Expanded analysis of iPSC astrocyte progenitor cytokine release profile.** Cytokines found present in all iPSC astrocyte progenitor-seeded scaffolds are displayed as % fold change relative to soft-only scaffolds.


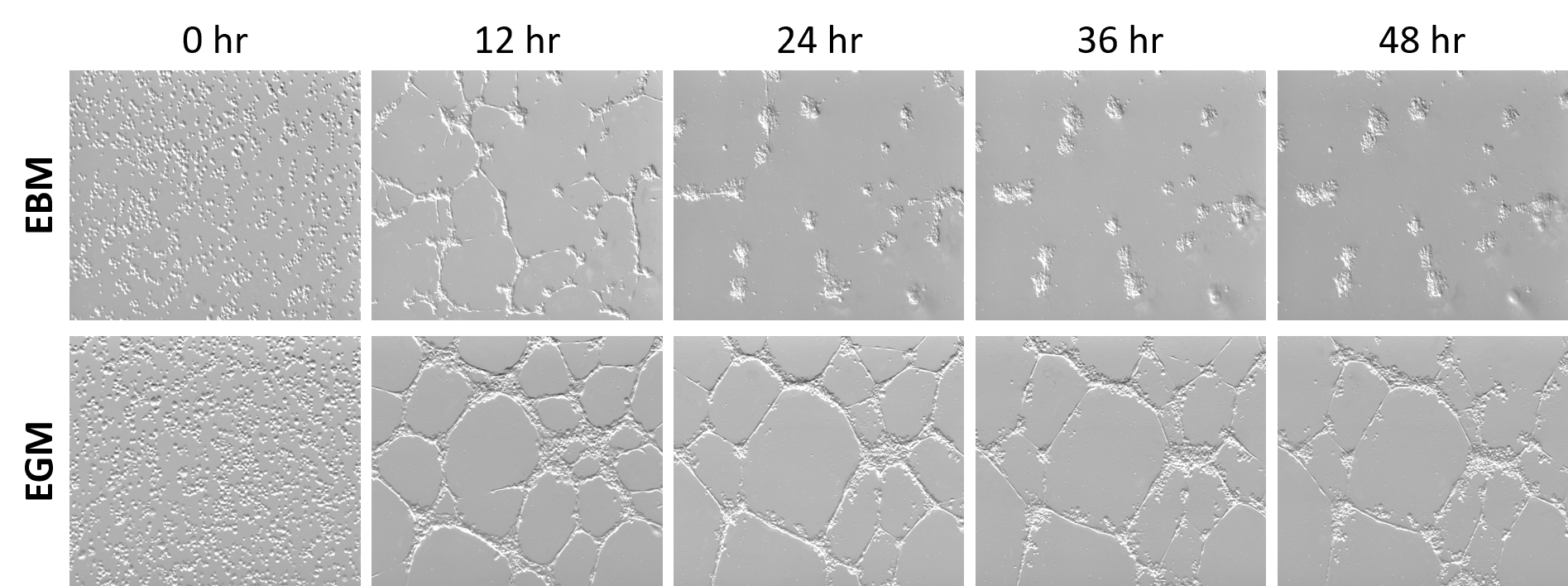


**Figure S5. Tube-formation assay control images**. Phase-contrast imaging shows the effect of endothelial basal media (EBM, negative control) and endothelial growth media (EGM, positive control) on endothelial tubule network formation over 48 hrs.


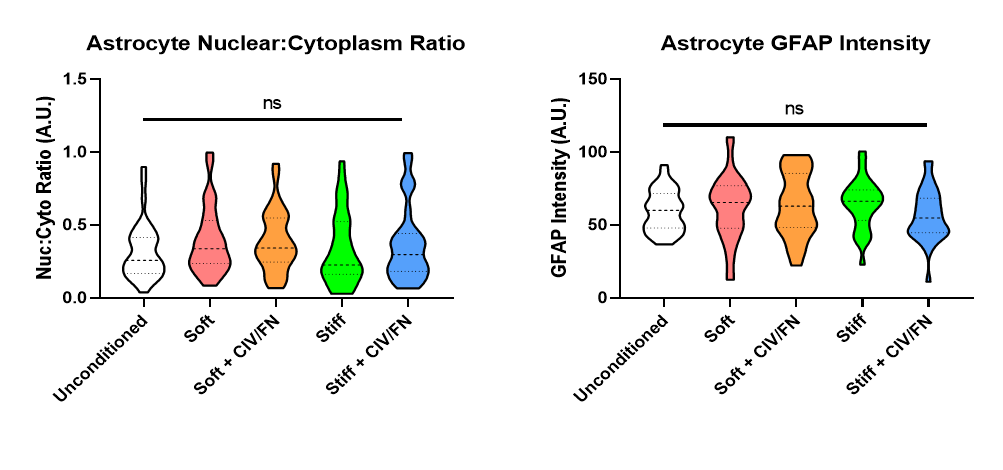


**Figure S6. Biomimetic iPSC astrocyte progenitor hyaluronic acid seeded scaffolds do not affect uninjured astrocyte reactivity.** A-B) Scaffold conditioned media treatments did not affect astrocyte reactive indexes of nuclear:cytoplasm ratio or GFAP intensity following 7 days of culture. N=3, n=34-39 fields of view. All analysis via one-way ANOVA and Tukey post-hoc test.


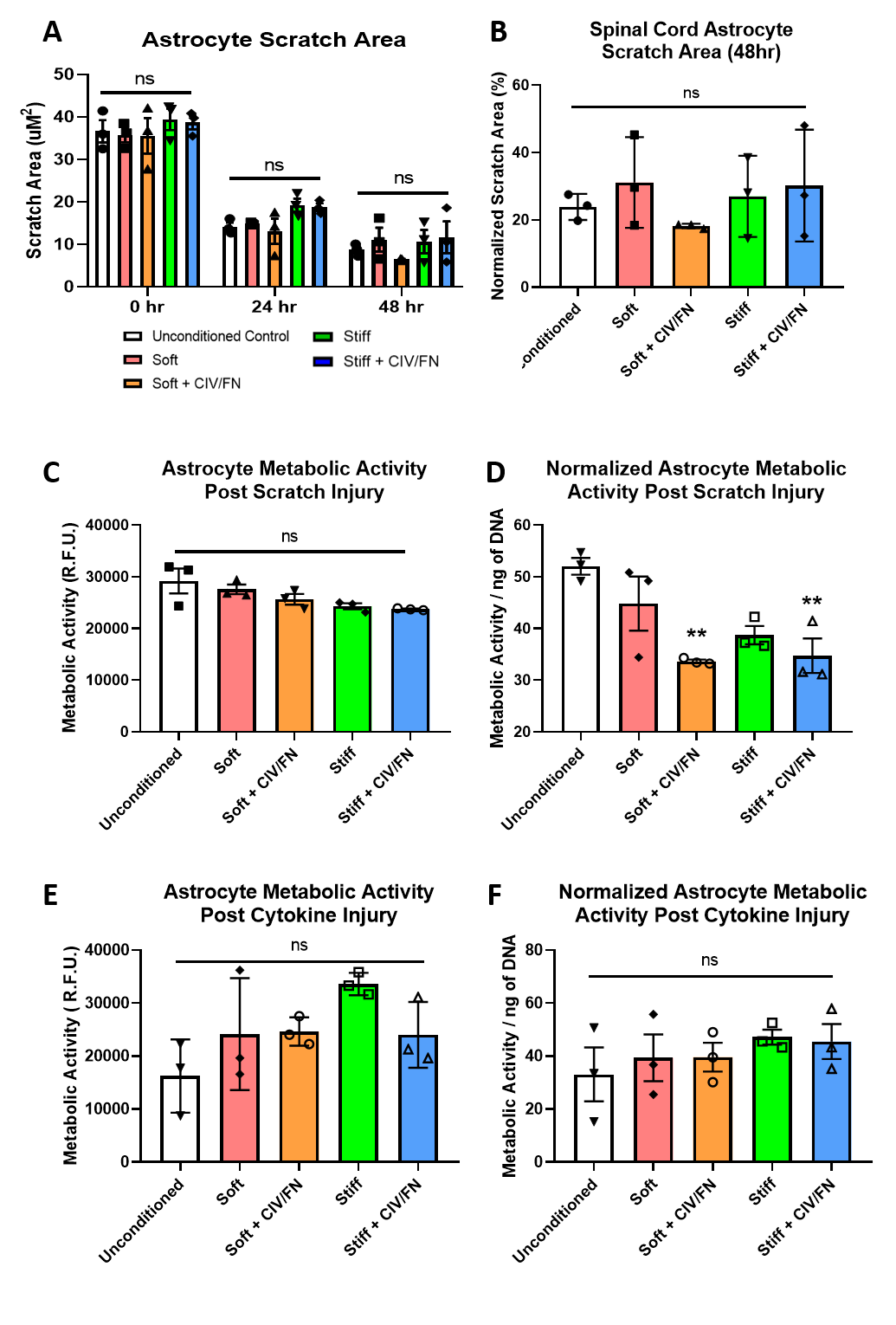


**Figure S7. Expanded analysis of spinal cord astrocyte injury response to IPSC astrocyte progenitor seeded scaffold conditioning**. A) No significant differences were noted in the scratch area at 24 or 48 hrs following all scaffold media treatments. B) Scratches exposed to media from iPSC astrocyte progenitor-seeded soft CIV/FN scaffold groups showed the largest decrease in scratch area at 48 hrs. C) Different scaffold conditioned groups did not affect overall astrocyte metabolic activity 48 hrs after scratch injury. D) Injured astrocytes conditioned by CIV/FN scaffold groups showed the lowest levels of metabolic activity normalized to DNA content (**p<0.01 vs unconditioned). E-F) No significant differences were detected between different media treatments for metabolic activity or metabolic activity normalized to DNA content following cytokine injury and scaffold conditioning. A) Analysis via two-way ANOVA, Bonferroni post hoc test. All other analyses were via one-way ANOVA, Tukey post hoc test. N=3.


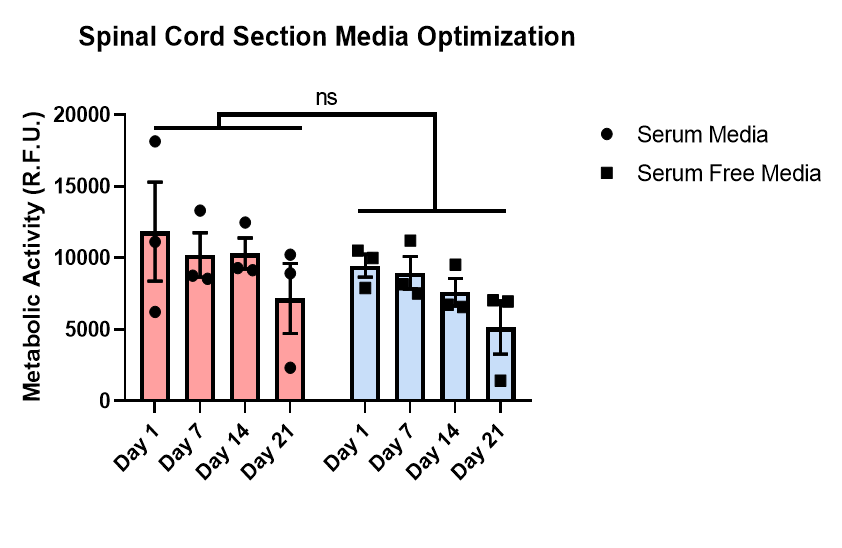


**Figure S8. Serum free media supports mouse spinal cord slice viability up to 21 days in culture.** Media optimization revealed that spinal explants cultured in iPSC astrocyte progenitor serum free media presented similar levels of viability to those cultured in serum media over 21 days.

**
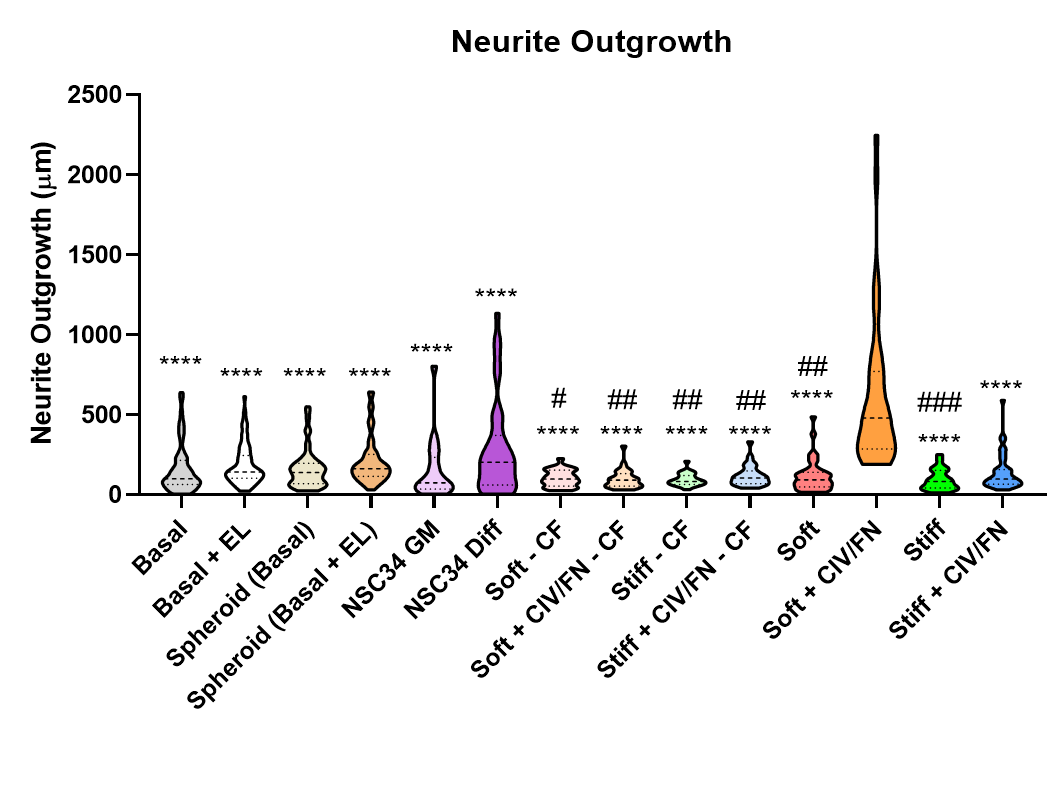
**

**Figure S9. Soft collagen-IV and fibronectin scaffolds enhanced the neurotrophic capacity of astrocyte progenitors to promote NSC-34 neuronal growth – extended analysis**. In addition to testing, iPSC astrocyte progenitor-seeded scaffold conditioned media treatments, further controls were tested to confirm the potency of the soft CIV/FN iPSC astrocyte progenitor-seeded scaffolds. Rationale & description for each group included below from left to right; (I) Astrocyte progenitor basal and basal media with differentiation factors epidermal growth factor and leukemia inhibitory factor (Basal + EL): To ensure differentiation factors of the unconditioned media iPSC astrocyte progenitor basal media were not affecting neurite extension; (II) IPSC astrocyte progenitor scaffold-free spheroids cultured in basal media and basal media with differentiation factors epidermal growth factor and leukemia inhibitory factor (Basal + EL): To determine whether progenitor spheres in scaffold-free, low adhesion culture plate conditions could promote neurite outgrowth; (III) NSC34 Growth media (GM): negative control for standard NSC 34 growth without differentiation factors; (IV) NSC 34 Diff media: Positive control containing neurogenic ATRA to observe neurite extension in conditions commonly used to promote extension; (V) Cell-free (CF) scaffold groups: Conditioned media from scaffolds without cells to ensure matrix degradation was not promoting neurite outgrowth. *vs soft + CIV/FN, #vs NSC 34 Differentiation. N=3, 24-72 fields of view. Analysis via one-way ANOVA & Tukey post-hoc.
